# Rasa3 Controls Megakaryocyte Rap1 Activation, Integrin Signaling and Differentiation into Proplatelet

**DOI:** 10.1371/journal.pgen.1004420

**Published:** 2014-06-26

**Authors:** Patricia Molina-Ortiz, Séléna Polizzi, Eve Ramery, Stéphanie Gayral, Céline Delierneux, Cécile Oury, Shintaro Iwashita, Stéphane Schurmans

**Affiliations:** 1 Laboratory of Functional Genetics, GIGA-Research Centre, Université de Liège, Liège, and Welbio, Belgium; 2 Institut de Recherches Interdisciplinaires en Biologie Humaine et Moléculaire (IRIBHM), Institut de Biologie et de Médecine Moléculaires (IBMM), Faculté de Médecine, Université Libre de Bruxelles, Gosselies, Belgium; 3 Laboratoire de Biologie Clinique, Faculté de Médecine-vétérinaire, Université de Liège, Liège, Belgium; 4 Laboratory of Thrombosis and Hemostasis, GIGA-Research Centre, Université de Liège, Liège, Belgium; 5 Mitsubishi Kagaku Institute of Life Sciences and Faculty of Pharmacy, Iwaki Meisei University, Iwaki, Japan; Centre for Cancer Biology, SA Pathology, Australia

## Abstract

Rasa3 is a GTPase activating protein of the GAP1 family which targets Ras and Rap1. Ubiquitous Rasa3 catalytic inactivation in mouse results in early embryonic lethality. Here, we show that Rasa3 catalytic inactivation in mouse hematopoietic cells results in a lethal syndrome characterized by severe defects during megakaryopoiesis, thrombocytopenia and a predisposition to develop preleukemia. The main objective of this study was to define the cellular and the molecular mechanisms of terminal megakaryopoiesis alterations. We found that Rasa3 catalytic inactivation altered megakaryocyte development, adherence, migration, actin cytoskeleton organization and differentiation into proplatelet forming megakaryocytes. These megakaryocyte alterations were associated with an increased active Rap1 level and a constitutive integrin activation. Thus, these mice presented a severe thrombocytopenia, bleeding and anemia associated with an increased percentage of megakaryocytes in the bone marrow, bone marrow fibrosis, extramedular hematopoiesis, splenomegaly and premature death. Altogether, our results indicate that Rasa3 catalytic activity controls Rap1 activation and integrin signaling during megakaryocyte differentiation in mouse.

## Author Summary

Megakaryocytes are the bone marrow cellular precursors of circulating blood platelets and give rise to nascent platelets by forming branching filaments called proplatelets. Terminal differentiation of round megakaryocytes into branched proplatelet forming megakaryocytes is a complex cytoskeletal-driven process which is affected in rare human familial thrombocytopenias. Interactions of megakaryocytes with extracellular matrix proteins are essential in this process since constitutive megakaryocyte integrin activity caused by specific mutations in ITGA2B or ITGB3 genes encoding for extracellular matrix protein receptors may result in abnormal adherent megakaryocytes, defect in proplatelet formation and thrombocytopenia. Here, we show that Rasa3, a GTPase activating protein of the GAP1 family, controls Rap1 activation and integrin signaling during megakaryocyte differentiation. We found that Rasa3 catalytic inactivation in mice altered megakaryocyte development, adherence, migration, actin cytoskeleton organization and differentiation into proplatelet. Thus, these mice presented a severe thrombocytopenia, bleeding and anemia.

## Introduction

Ras families GTPase-activating proteins (GAP), like Ras GAPs, Rho GAPs and Arf GAPs, are tumor suppressors as the loss of their GAP activity allows uncontrolled Ras, Rho and Arf activities and promotes cancer. Rasa3 (or GAP1^IP4BP^, R-Ras GAP) is a member of the Ras GAP1 subfamily with Rasa2 (or GAP1^m^), Rasa4 (or Capri) and Rasal (or Rasal1) [1]–[5]. This Ras GAP subfamily is known to function as dual GAP for Ras an Rap-GTPases [6], [7]. Rasa3 protein structure is characterized by a conserved basic domain structure comprising two N-terminal tandem C2 domains, a central GAP domain and a C-terminal pleckstrin homology (PH) domain that is associated with a Bruton's tyrosine kinase (Btk) motif [8]. Binding of the latter domain to phosphoinositides determines Rasa3 targeting to the cytosolic leaflet of the plasma membrane where it inactivates Ras and Rap1 [9]–[11]. Down-regulation of Rasal and Rasa4 induces cellular transformation *in vitro* [12], [13], and Rasal is down-regulated in multiple human tumors by epigenetic silencing [14]. Rasa4 inactivation in mouse leads to impaired macrophages Fcγ receptor-mediated phagocytosis and oxidative burst, as well as to increased bacterial infection [15]. No clear definition of Rasa2 function *in vivo* is currently available. Mutant mice expressing a catalytically-inactive Rasa3 protein have been reported to die at mid embryonic life [16]. Indeed, removal of exons 11 and 12 of the mouse Rasa3 gene, 2 exons which are essential for the Ras GAP activity, leads to the expression of a 88 amino acids-truncated but catalytically inactive Rasa3 protein [16]. Phenotypically, Rasa3 mutant embryos present massive subcutaneous and intraparenchymal hemorrhages probably consecutive to abnormal adherens junctions between capillary endothelial cells [16]. Multiple roles for Ras and Rap1, the Rasa3 targets, have been defined in hematopoietic cells: these proteins control cellular proliferation, differentiation, migration and adhesion. In particular, Rap1 has been implicated in the maturation of megakaryocytes and the pathogenesis of chronic myelogenous leukemia [17]. Here, we found that catalytic inactivation of Rasa3 specifically in the hematopoietic system results in a lethal syndrome characterized by major alterations during megakaryopoiesis. These alterations were associated with increased active Rap1 level and constitutive integrin activation in megakaryocytes, a phenotype quite different clinically, biologically and mechanistically from that of recently published mice with a spontaneous missense mutation between the two N-terminal tandem C2 domains of Rasa3 [18].

## Results

### The SCID-Rasa3 model

In order to study the specific effects of a catalytically-inactive Rasa3 mutant protein on the hematopoietic system and to circumvent the early embryonic lethality reported in Rasa3^−/−^ mice, we used irradiated Severe Combined Immune Deficient (SCID) mice reconstituted with E12.5 liver cells derived from Rasa3^+/+^, Rasa3^+/−^ or Rasa3^−/−^ embryos. SCID mice were first analyzed 6 weeks after irradiation/reconstitution: all Rasa3 genotypes were able to reconstitute the lymphoid compartment in irradiated SCID mice since no significant difference was detected between SCID-Rasa3^+/+^, SCID-Rasa3^+/−^ and SCID-Rasa3^−/−^ mice in total numbers of splenic T and B cells (Table S1). No significant difference was observed in red blood cell, blood platelet and bone marrow megakaryocyte counts as well as spleen weight between SCID-Rasa3^+/+^ and SCID-Rasa3^−/−^ mice at this stage (Table S1).

### Decreased survival, hemorrhages and splenomegaly in SCID-Rasa3^−/−^ mice

**Figure 1:**
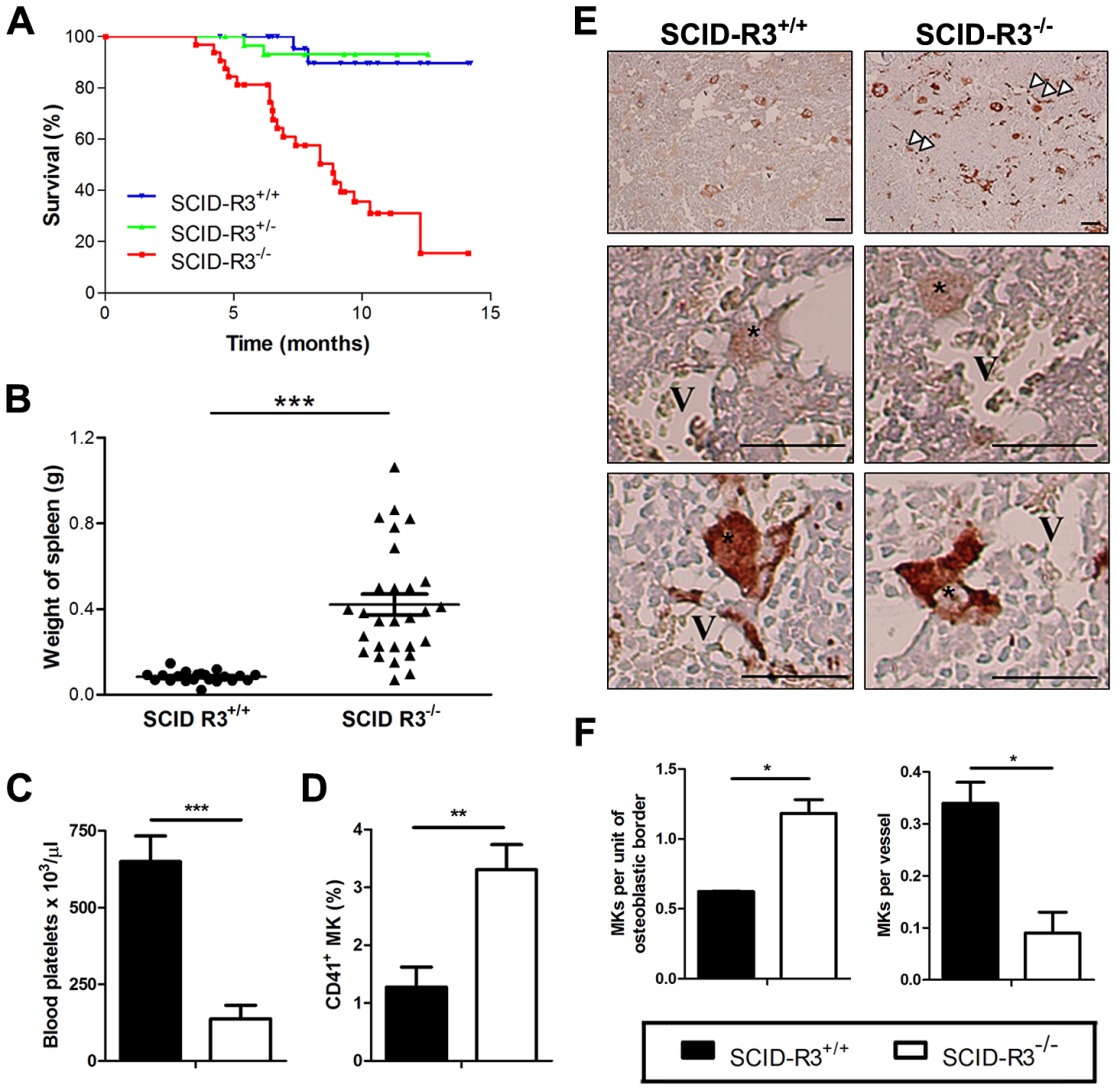
Decreased survival, splenomegaly, thrombocytopenia and megakaryocyte alterations in SCID-Rasa3^−/−^ mice. **A.** Survival of irradiated SCID mice reconstituted with Rasa3^+/+^ (SCID-R3^+/+^, n = 28 mice), Rasa3^+/−^ (SCID-R3^+/−^, n = 31 mice) or Rasa3^−/−^ (SCID-R3^−/−^, n = 32 mice) embryonic liver cells. **B.** Spleen weight from age-matched SCID-Rasa3^+/+^ and moribund SCID-Rasa3^−/−^ mice. The mean ± SEM are also presented in each group of mice. A splenomegaly was defined as a spleen weight over 0.168 g (i.e. twice the mean spleen weight of SCID-Rasa3^+/+^ mice). **C.** Blood platelet counts in age-matched SCID-Rasa3^+/+^ (black column, n = 9) and moribund SCID-Rasa3^−/−^ (white column, n = 15) mice. Results represent the mean ± SEM of platelets per µl of blood. **D.**Mean ± SEM of CD41^+^ megakaryocyte (MK) percentages detected by flow cytometry in the bone marrow isolated from age-matched SCID-Rasa3^+/+^ (black column, n = 17) and moribund SCID-Rasa3^−/−^ (white column, n = 20) femurs. **E.** vWF-stained bone marrow sections of SCID-Rasa3^+/+^ and SCID-Rasa3^−/−^ femurs 3 months after SCID mice reconstitution. V: vessel; *: megakaryocyte; arrowheads: abnormal vWF deposits. Scale bars: 50 µm. **F.** Quantification of megakaryocytes (MKs) in the osteoblastic and the vascular niches of SCID-Rasa3^+/+^ (black columns) and SCID-Rasa3^−/−^ (white columns) bone marrow femurs 3 months after irradiation/reconstitution. Results represent the mean ± SEM of the number of megakaryocytes per unit of osteoblastic border, or per vessel. Statistics (unpaired *t* test): *: P<0.05; **: P<0.01; ***: P<0.001.

More than 80% of SCID mice reconstituted with Rasa3^−/−^ cells died within 14 months after reconstitution while, at the same time, about 95% of SCID-Rasa3^+/+^ and SCID-Rasa3^+/−^ mice were still alive (Fig. 1a). Pathological analysis revealed that 85% of SCID-Rasa3^−/−^ mice presented with thoracic and/or peritoneal hemorrhages (data not shown) and that more than 80% had a splenomegaly (Fig. 1b). Our results below present the analysis of a total of 24 moribund SCID-Rasa3^−/−^ mice. Among these 24 mice, 20 had a megakaryocytic dysplasia associated with a severe thrombocytopenia, and the remaining 4 developed a preleukemia. The main objective of this study was to define the cellular and the molecular mechanisms of the megakaryocytic dysplasia.

### Thrombocytopenia and megakaryocyte alterations in 20/24 SCID-Rasa3^−/−^ mice

In ∼80% (20/24) of SCID-Rasa3^−/−^ mice, blood analysis revealed a thrombocytopenia (Fig. 1c). Thrombocytopenia in these mice was associated with megakaryocyte alterations. A significant increase in the percentage of CD41^+^ megakaryocytes was observed in the bone marrow of SCID-Rasa3^−/−^ mice (Fig. 1d). These megakaryocytes were morphologically abnormal and presented a marked increase in the intensity of von Willebrand factor (vWF) staining, as compared with SCID-Rasa3^+/+^ megakaryocytes (Fig. 1e). vWf^+^ deposits were also abnormally detected along the SCID-Rasa3^−/−^ diaphysis (Fig. 1e, arrowheads). In addition, more megakaryocytes were present in the osteoblastic niche in SCID-Rasa3^−/−^ mice, as compared with SCID-Rasa3^+/+^ mice (Fig. 1f, left panel). Inversely, the vascular niche hosted less megakaryocytes in mutant mice (Fig. 1f, right panel). The stem and megakaryocyte progenitor cell compartments were characterized in the bone marrow of SCID-Rasa3^+/+^ and SCID-Rasa3^−/−^ mice by flow cytometry. A similar percentage of live c-Kit^+^/Lin^−^ cells was detected in the bone marrow of these mice, and the proportion of Sca-1^+^ cells within this population was also not significantly different in SCID-Rasa3^+/+^ and SCID-Rasa3^−/−^ mice (Table S2). Staining of these c-Kit^+^ Lin^−^ Sca-1^+^ (KLS) cells with CD34 and Flk-2 antibodies defined the KLS-CD34^−^ Flk-2^−^ hematopoietic stem cell compartment; but again, no difference was detected in the percentage of these cells between SCID-Rasa3^+/+^ and SCID-Rasa3^−/−^ mice (Table S2). It has been recently shown that bone marrow progenitors with megakaryocyte potential reside in the Lin^−^ c-Kit^+^ FcγRII/III^lo^ Sca-1^−^ CD150^+^ cell population [19]. This cell population was decreased in the bone marrow of SCID-Rasa3^−/−^ mice, as compared with SCID-Rasa3^+/+^, but the difference did not reach statistical significance (Table S2).

Femur sections revealed the presence of a fibrosis characterized by numerous collagen trabeculae in the cavity of SCID-Rasa3^−/−^ femurs, while the cavity of SCID-Rasa3^+/+^ femurs was totally free of collagen trabeculae (Fig. S1a). Consequently, the number of nucleated cells recovered on average from one femur of SCID-Rasa3^−/−^ mice was significantly reduced, as compared with SCID-Rasa3^+/+^ mice (SCID-Rasa3^+/+^: 20.9±2.8×10^6^ nucleated cells, n = 12; SCID-Rasa3^−/−^: 6.4±1.1×10^6^ nucleated cells, n = 19; P<0.001, unpaired *t* test). Bone marrow cell density was similar in the cavity of SCID-Rasa3^+/+^ and SCID-Rasa3^−/−^ femurs (Fig. S1b), and similar percentages of CD117^+^/c-Kit^+^ progenitor cells and Ter119^+^ CD71^+^ erythroblasts were detected in SCID-Rasa3^+/+^ and SCID-Rasa3^−/−^ bone marrow cells (Table S2 and data not shown). An increased splenic hematopoiesis - including megakaryopoiesis -, associated with a disorganized pulp architecture, and foci of liver hematopoiesis were also observed in SCID-Rasa3^−/−^ mice (Table S3 and Fig. S2).

Thrombocytopenia and hemorrhages in SCID-Rasa3^−/−^ mice were associated with a regenerative anemia, whereas normal counts were maintained for total white blood cell and circulating neutrophil, lymphocyte, monocyte and eosinophil (Fig. S3 and Table S4). Thrombopoietin (TPO) levels were significantly decreased in SCID-Rasa3^−/−^ mice, as compared with SCID-Rasa3^+/+^ mice, a probable consequence of the markedly increased Mpl^+^/CD150^+^ megakaryocyte number in the spleen of these mice (TPO level in SCID-Rasa3^+/+^ mice: 1732±211 pg/ml, n = 11; TPO level in SCID-Rasa3^−/−^ mice: 653±74 pg/ml, n = 16; mean ± SEM; P<0.001).

Collectively, our results indicate that the loss of Rasa3 catalytic activity in 20/24 SCID-Rasa3^−/−^ mice leads to megakaryocyte alterations, to thrombocytopenia, hemorrages and a regenerative anemia.

### Altered megakaryocyte adhesion, motility and capacity to differentiate in proplatelet forming megakaryocytes in SCID-Rasa3^−/−^ mice

**Figure 2:**
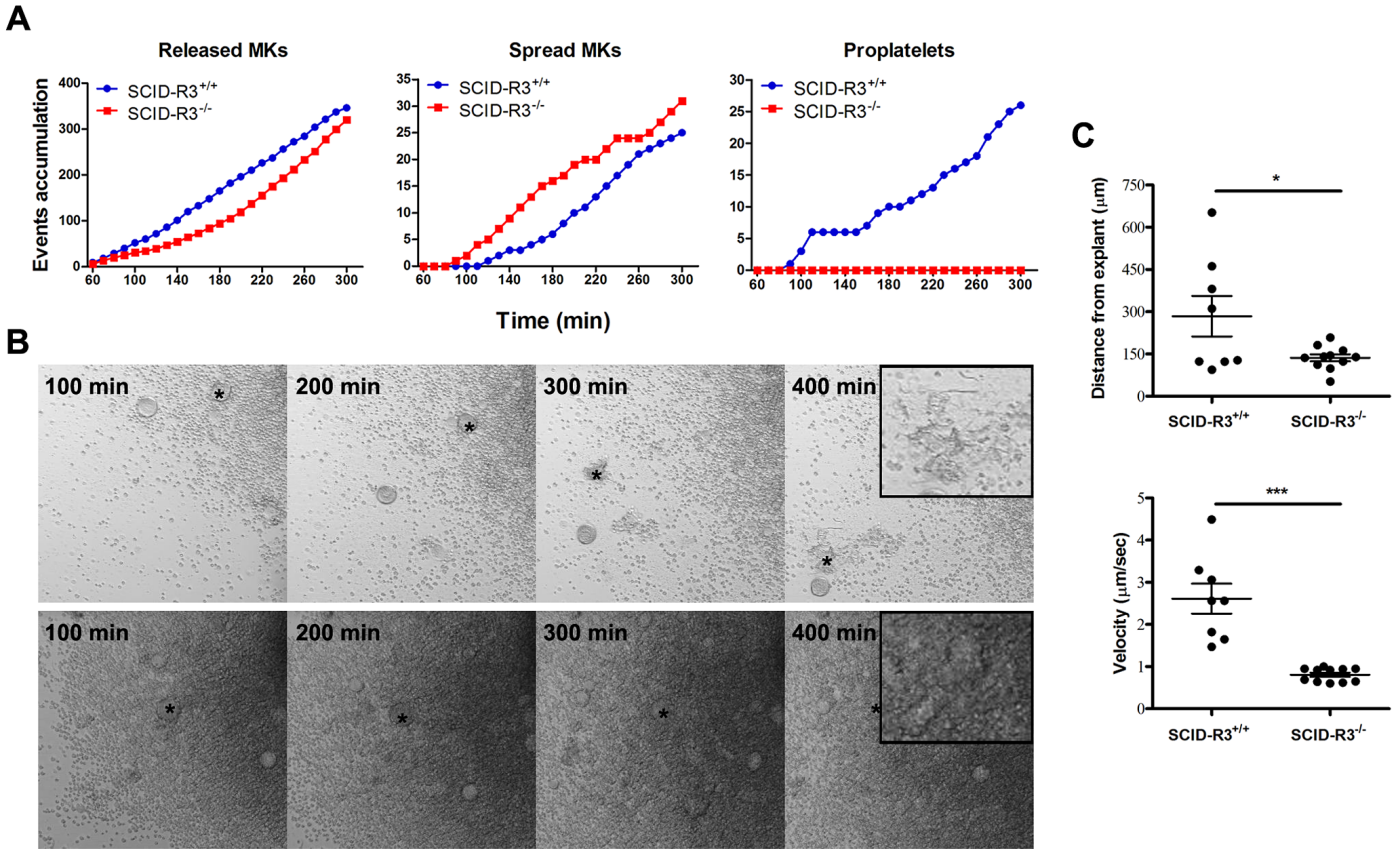
Altered SCID-Rasa3^−/−^ megakaryocyte motility, adhesion and differentiation into proplatelet forming megakaryocytes. Femur bone marrow explants were isolated from SCID-Rasa3^+/+^ and SCID-Rasa3^−/−^ mice 2 months after irradiation/reconstitution and cultured under a confocal microscope. Images were taken in bright field every 10 min. **A.** Number of megakaryocytes released from the explants (left panel), of spreading megakaryocytes (center panel) and of proplatelet forming megakaryocytes (right panel). Results are representative of 3 independent experiments. **B.** Example of megakaryocytes released from a SCID-Rasa3^+/+^ (upper panels) and a SCID-Rasa3^−/−^ (lower panels) explant. The asterisk indicates the same megakaryocyte that finally develops into a proplatelet forming megakaryocyte (SCID-Rasa3^+/+^ explants) or that continuously adheres to the culture plate and fail to form proplatelets (SCID-Rasa3^−/−^ explants). Insets: higher magnification shows the proplatelet forming megakaryocyte in the SCID-Rasa3^+/+^ explants and the adherent megakaryocyte in the SCID-Rasa3^−/−^ explants. Scale bars: 50 µm. **C.** Velocity and linear distance covered by individual megakaryocytes 3 hours after release from SCID-Rasa3^+/+^ and SCID-Rasa3^−/−^ explants. Statistics (unpaired *t* test): *: P<0.05; ***: P<0.001.

Bone marrow was isolated from SCID-Rasa3 mice 2 months after irradiation/reconstitution and cultured under a confocal microscope. Despite a ∼2-fold increased percentage of megakaryocytes in the SCID-Rasa3^−/−^ bone marrow, there was a trend for a decreased number of megakaryocytes released from SCID-Rasa3^−/−^ bone marrow explants, as compared with SCID-Rasa3^+/+^ explants (Fig. 2a, left panel). Released SCID-Rasa3^−/−^ megakaryocytes were able to spread on the culture plate but never fully differentiated in proplatelet forming megakaryocytes (Fig. 2a, centre and right panels, and Fig. 2b). The linear distance covered by the released megakaryocytes and their velocity were significantly lower in SCID-Rasa3^−/−^ than in SCID-Rasa3^+/+^ explants (Fig. 2c).

### Altered actin cytoskeletal organization in Rasa3^−/−^ adherent megakaryocytes

**Figure 3:**
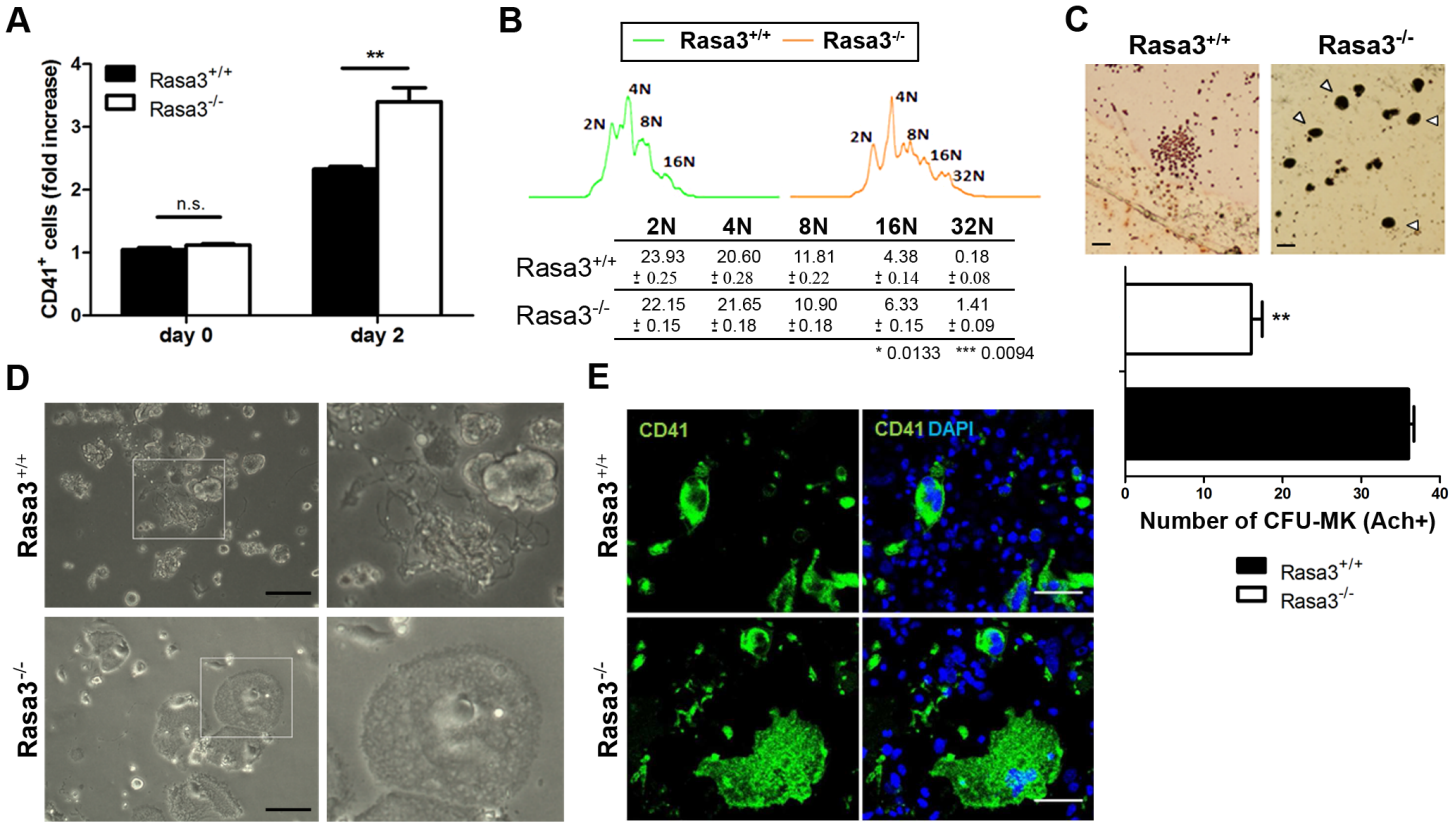
Abnormal megakaryocyte differentiation from Rasa3^−/−^ fetal liver cell culture. Fetal liver cell (FLC) were isolated from E12.5 Rasa3^+/+^ and Rasa3^−/−^ embryos. **A**. Percentages of CD41^+^ megakaryocytes were determined at day 0 and 2 days after TPO treatment in 6 Rasa3^+/+^ and 8 Rasa3^−/−^ FLC cultures by flow cytometry. Results are expressed as fold of increase of CD41^+^ cells, considering the percentage of Rasa3^+/+^ CD41^+^ cells at day 0 as 1. Statistics (unpaired *t* test): **: P<0.01. **B**. Increased megakaryocyte ploidy in FLC culture at day 2 after TPO treatment. Representative images of DNA content in Rasa3^+/+^ and Rasa3^−/−^ CD41^+^ megakaryocytes. The table shows a quantification of the percentages of individual ploidy classes of FLC-derived CD41^+^ megakaryocytes (mean ± SEM). Statistics (unpaired *t* test, n = 6): *: P<0.013; ***: P<0.009. **C**. CFU-Mk assay from Rasa3^+/+^ and Rasa3^−/−^ FLC. Representative images of a Rasa3^+/+^ CFU-Mk with immature megakaryocytes of about 10 µm of diameter, and of Rasa3^−/−^ mature megakaryocytes of about 30 µm of diameter (arrowheads). Scale bars: 50 µm. The graph represents the number of CFU-Mk formed by 5 Rasa3^+/+^ and 3 Rasa3^−/−^ independent FLC cultures after 3 days (mean ± SEM). Statistics (unpaired *t* test): **: P<0.01. **D.** Representative images of FLC Rasa3^+/+^ (upper panels) and Rasa3^−/−^ (lower panels) cultures after 6 days with TPO. A digital magnification (3×) of a proplatelet event in Rasa3^+/+^ FLC culture (right upper panel) and of an abnormal adherent megakaryocyte in Rasa3^−/−^ FLC culture (right lower panel) are presented. In Rasa3^+/+^ FLC culture, 8.4±2.2% of megakaryocytes formed proplatelets. No proplatelet forming megakaryocyte was detected in Rasa3^−/−^ FLC culture. Scale bars: 50 µm. **E.** Rasa3^+/+^ (upper panels) and Rasa3^−/−^ (lower panels) FLC cultures after 6 days with TPO were stained with a CD41 antibody (green, left panels) or CD41 and DAPI (green and blue, respectively; right panels). The large abnormal adherent cell population detected in Rasa3^−/−^ FLC culture is CD41-positive. In Rasa3^+/+^ FLC culture, 8.4±2.2% of megakaryocytes formed proplatelets. No proplatelet forming megakaryocyte was detected in Rasa3^−/−^ FLC culture. Scale bars: 50 µm.

In order to further analyze the role of Rasa3 in megakaryocyte adhesion and differentiation, we used megakaryocytes obtained from Rasa3^+/+^ and Rasa3^−/−^ fetal liver cells (FLC) cultured in the presence of TPO. This cellular model is simpler and faster than the model of bone marrow explants isolated from SCID-Rasa3 mice, and it recapitulated the megakaryocyte defects previously observed in the later model. Indeed, after 2 days of TPO treatment, flow cytometry analysis detected a significant 1.42-fold increase in the percentage of CD41^+^ megakaryocytes in the Rasa3^−/−^ FLC culture, as compared with Rasa3^+/+^ culture (Fig. 3a). CD41^+^ megakaryocytes with 16N and 32N ploidy were significantly increased in these Rasa3^−/−^ FLC cultures, as compared with Rasa3^+/+^ FLC cultures (Fig. 3b). Colony-forming unit-megakaryocyte (CFU-Mk) assay using FLC revealed a significant decrease in the number of small immature megakaryocyte colonies in Rasa3^−/−^ cell culture, as compared with Rasa3^+/+^ cell culture (Fig. 3c). However, numerous large mature megakaryocytes were detected in the Rasa3^−/−^ CFU-Mk assay, while not in the Rasa3^+/+^ CFU-Mk assay (Fig. 3c, arrowheads). After 6 days of TPO treatment, many proplatelets were observed in the Rasa3^+/+^ FLC culture whereas, in the Rasa3^−/−^ FLC culture, no proplatelets were detected (Fig. 3d). Instead of proplatelets, many abnormal adherent cells were detected in the Rasa3^−/−^ FLC culture that were CD41 positive, indicating their megakaryocyte origin (Fig. 3d, lower panels and Fig. 3e).

**Figure 4:**
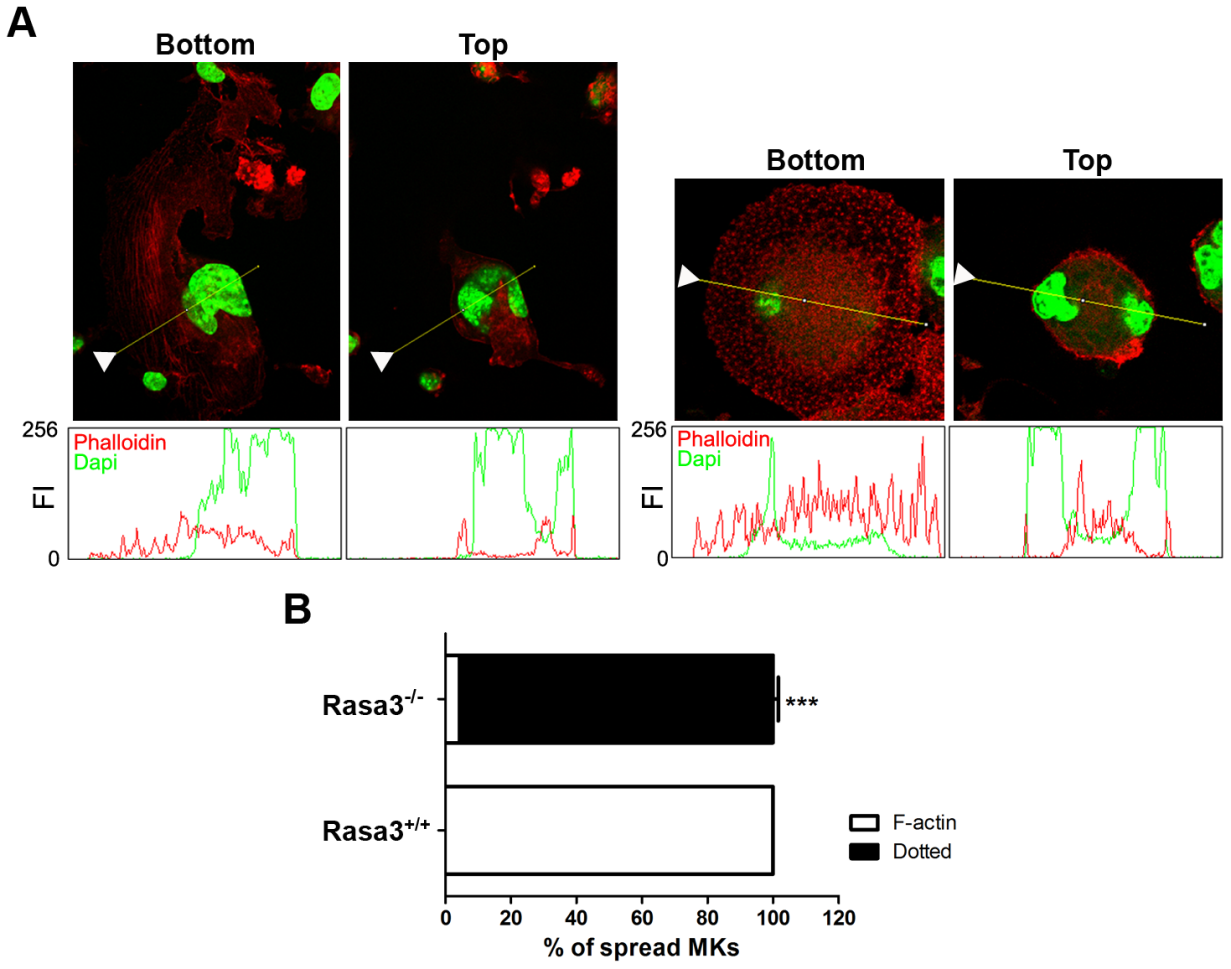
Abnormal actin cytoskeleton organization in megakaryocytes derived from Rasa3^−/−^ fetal liver cell culture. Fetal liver cell (FLC) were isolated from E12.5 Rasa3^+/+^ and Rasa3^−/−^ embryos and cultured for 6 days with TPO. **A**. Rasa3^+/+^ (left bottom and top panels) and Rasa3^−/−^ (right bottom and top panels) adherent megakaryocytes in FLC culture after staining with phalloidin-TRITC (actin, red) and DAPI (green). Confocal images were obtained at the bottom and the top of the same megakaryocyte. Graphs represent the fluorescence intensity (FI, scaled from 0 to 256) of phalloidin (actin) and DAPI stainings along the indicated line. The arrowheads on the images indicated the origin of the line. Twenty megakaryocytes were analyzed in duplicate per FLC culture, 6 Rasa^+/+^ and 5 Rasa3^−/−^ FLC cultures. Representative images are shown. **B**. Quantification of the percentage of adherent Rasa3^+/+^ and Rasa3^−/−^ megakaryocytes with a dotted (ie when F-actin is not detected) or a F-actin (ie when stress fiber can be detected) phenotype (mean ± SEM). Fifty megakaryocytes were analyzed in duplicate per FLC culture, 12 Rasa3^+/+^ and 10 Rasa3^−/−^. Statistics (unpaired *t* test): ***: P<0.001.

Actin cytoskeleton staining of these abnormal adherent Rasa3^−/−^ megakaryocytes revealed a unique dotted actin pattern without stress fiber at the contact with the culture plate, significantly different from the expected actin stress fiber pattern observed in the few adherent Rasa3^+/+^ megakaryocytes present in the FLC culture at day 6 of TPO treatment (Fig. 4a, bottom, and 4b). The actin cytoskeletal organization was also altered at the top of the adherent Rasa3^−/−^ megakaryocytes: actin was decreased at the periphery and much more concentrated at the center of the cell, as compared with adherent Rasa3^+/+^ megakaryocytes (Fig. 4a, top).

Collectively, these results indicate that Rasa3^−/−^ FLC abnormally develop into mature megakaryocytes, and that Rasa3^−/−^ megakaryocytes derived from FLC culture have an altered actin cytoskeleton organization associated with an abnormal adherent phenotype, a reduced motility and an absence of normal terminal differentiation in proplatelets. Interestingly, this Rasa3^−/−^ megakaryocyte phenotype (i.e. defect in proplatelet formation, dotted actin cytoskeletal pattern with reduced stress fibers and abnormal adherent megakaryocytes) resembles that of rare thrombocytopenic patients with a constitutive αIIbβ3 integrin activity caused by specific mutations in ITGA2B or ITGB3 genes [20]–[22].

### Altered inside-out and outside-in integrin signaling in Rasa3^−/−^ megakaryocytes

**Figure 5:**
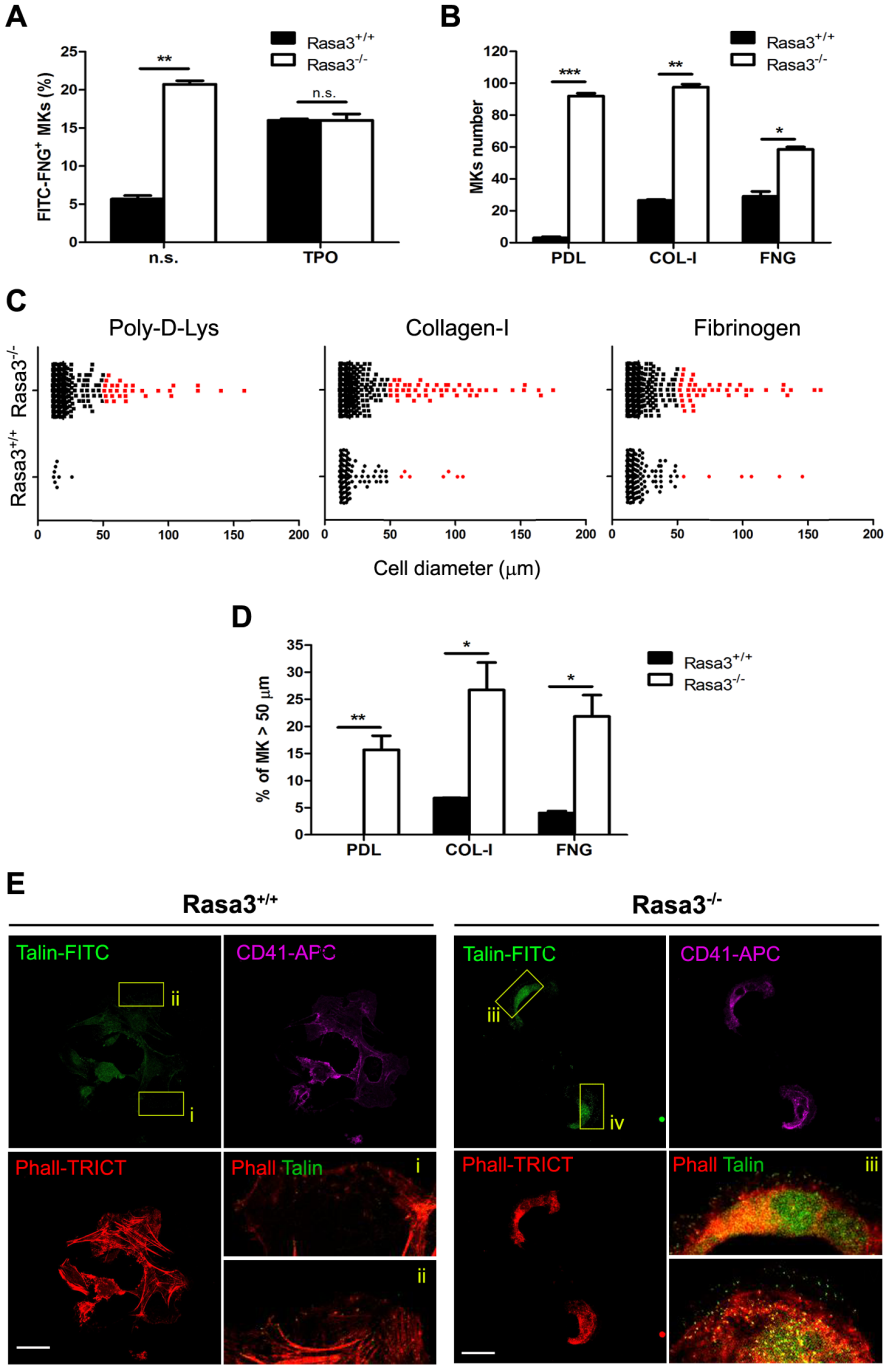
Altered inside-out and outside-in integrin signaling in Rasa3^−/−^ megakaryocytes. Fetal liver cell (FLC) were isolated from E12.5 Rasa3^+/+^ and Rasa3^−/−^ embryos and cultured with TPO for 3 days. Megakaryocytes were enriched on a BSA-gradient, deprived of serum for 4 hours and used in inside-out and outside-in integrin signaling assays. **A.** Inside-out αIIbβ3 integrin signaling was investigated in megakaryocytes by quantifying soluble FITC-fibrinogen (FITC-FNG) bound to the CD41^+^ cell surface by flow cytometry. Megakaryocytes were treated with or without 100 ng/ml TPO for 30 min. Specific binding was obtained after subtraction of the amount of soluble fibrinogen bound to the cell surface in the presence of EDTA and was expressed relative to the maximum binding obtained in the presence of MnCl_2_. A. U.: arbitrary units. n. s.: non stimulated. **B.**, **C.**, **D.** and **E.** Megakaryocytes were incubated for 18 hours on Poly-D-Lysine- (PDL), collagen-I- (COL-I) and fibrinogen- (FNG) coated plates in medium containing 10% FBS. Number (**B**, mean ± SEM) and diameter (**C**) of adherent megakaryocytes (MKs) was determined in 16 fields. Results are representative of 3 independent experiments. **D**. The percentage of Rasa3^−/−^ adherent megakaryocytes with a diameter over 50 µm was significantly increased, as compared with Rasa3^+/+^ megakaryocytes (mean ± SEM of 3 independent experiments). **E.** Rasa3^+/+^ and Rasa3^−/−^ PDL-adherent megakaryocytes were stained with phalloidin-TRICT (actin, red), CD41-APC (magenta) and Talin-FITC (green). Confocal images were obtained from the bottom of the cells. (i–iv): 4× Digital magnification of phalloidin-TRICT and Talin-FITC merge. An increased Talin staining is observed in Rasa3^−/−^ megakaryocytes (iii and iv), as compared with Rasa3^+/+^ megakaryocytes (i and ii). Scale bar: 50 µm. Statistics (unpaired *t* test): *: P<0.05; **: P<0.01; ***: P<0.001.

Soluble fibrinogen binding to αIIbβ3 integrin present at the megakaryocyte surface is regulated by inside-out signaling which determines the affinity/avidity of the integrin for its ligand. In the absence of megakaryocyte stimulation, only little amount of soluble FITC-fibrinogen bound to day 3 FLC culture-derived Rasa3^+/+^ mature megakaryocytes (Fig. 5a). By contrast, in this resting condition, a larger amount of soluble FITC-fibrinogen bound to Rasa3^−/−^ mature megakaryocytes, reaching the binding level of Rasa3^+/+^ megakaryocytes when stimulated by TPO for 30 min (Fig. 5a). Stimulation of Rasa3^−/−^ mature megakaryocytes by TPO did not further increase soluble FITC-fibrinogen binding. Importantly, no difference in αIIb/CD41 surface expression was detected by flow cytometry between day 3 FLC-derived Rasa3^+/+^ and Rasa3^−/−^ mature megakaryocytes (Rasa3^+/+^: 1234±70 arbitrary units (A. U.), Rasa3^−/−^: 1084±244 A. U., n = 3 independent experiments, P = 0.11), suggesting that Rasa3^−/−^ megakaryocytes have a constitutively activated inside-out signaling leading to a constitutive binding of soluble fibrinogen to αIIbβ3 integrin. Staining of day 3 FLC culture-derived Rasa3^+/+^ and Rasa3^−/−^ mature megakaryocytes with the JON/A antibody, which selectively binds to the high affinity conformation of integrin αIIbβ3, confirmed this hypothesis: a significant increase of JON/A^+^ megakaryocyte percentage was detected in Rasa3^−/−^ megakaryocytes in resting and TPO-stimulated conditions, as compared with Rasa3^+/+^ megakaryocytes (percentage of JON/A^+^ megakaryocytes, mean ± SEM: non-stimulated Rasa3^+/+^ megakaryocytes: 14.0±1.9%; non-stimulated Rasa3^−/−^ megakaryocytes: 70.1±3.4%, P = 0.002; TPO-stimulated Rasa3^+/+^: 23.3±0.5%; TPO-stimulated Rasa3^−/−^ megakaryocytes: 73.1±3.4%, P = 0.02).

Integrin activation triggers megakaryocyte adhesion to immobilized integrin ligands like collagen-I or fibrinogen and an outside-in signaling, resulting in the reorganization of the actin filaments and the modification of the cell shape [23]. Megakaryocytes from FLC-Rasa3^+/+^ cultured on day 3 adhered to collagen-I- and fibrinogen-coated plates, but nearly not to Poly-D-Lysine-coated plates, as expected (Fig. 5b). Adherence to immobilized collagen-I and fibrinogen resulted in cell spreading reaching diameters over 50 µm in a limited number of Rasa3^+/+^ megakaryocytes, as described (Fig. 5c, red dots, and Fig. 5d) (24). Adherence to Poly-D-Lysine-, collagen-I- and fibrinogen-coated plates was significantly higher in Rasa3^−/−^ than in Rasa3^+/+^ megakaryocytes (Fig. 5b). The percentage of megakaryocytes with a diameter over 50 µm was significantly increased in the Rasa3^−/−^ culture, as compared with the Rasa3^+/+^ culture (Fig. 5d). Outside-in integrin activation triggers the binding of the cytoskeletal protein talin to membrane integrins [24]. In association with their abnormal adhesion properties, Poly-D-Lysine adherent Rasa3^−/−^ megakaryocyte recruited more talin to their membrane, as compared with Rasa3^+/+^ megakaryocytes (Fig. 5e)

Collectively, our results indicate that Rasa3^−/−^ megakaryocytes have a constitutively activated inside-out αIIbβ3 integrin signaling associated with major alterations in outside-in integrin signaling leading to cell adherence and spreading independently of integrin ligands.

### Increased active GTP-bound Rap1 in Rasa3^−/−^ adherent megakaryocytes

**Figure 6:**
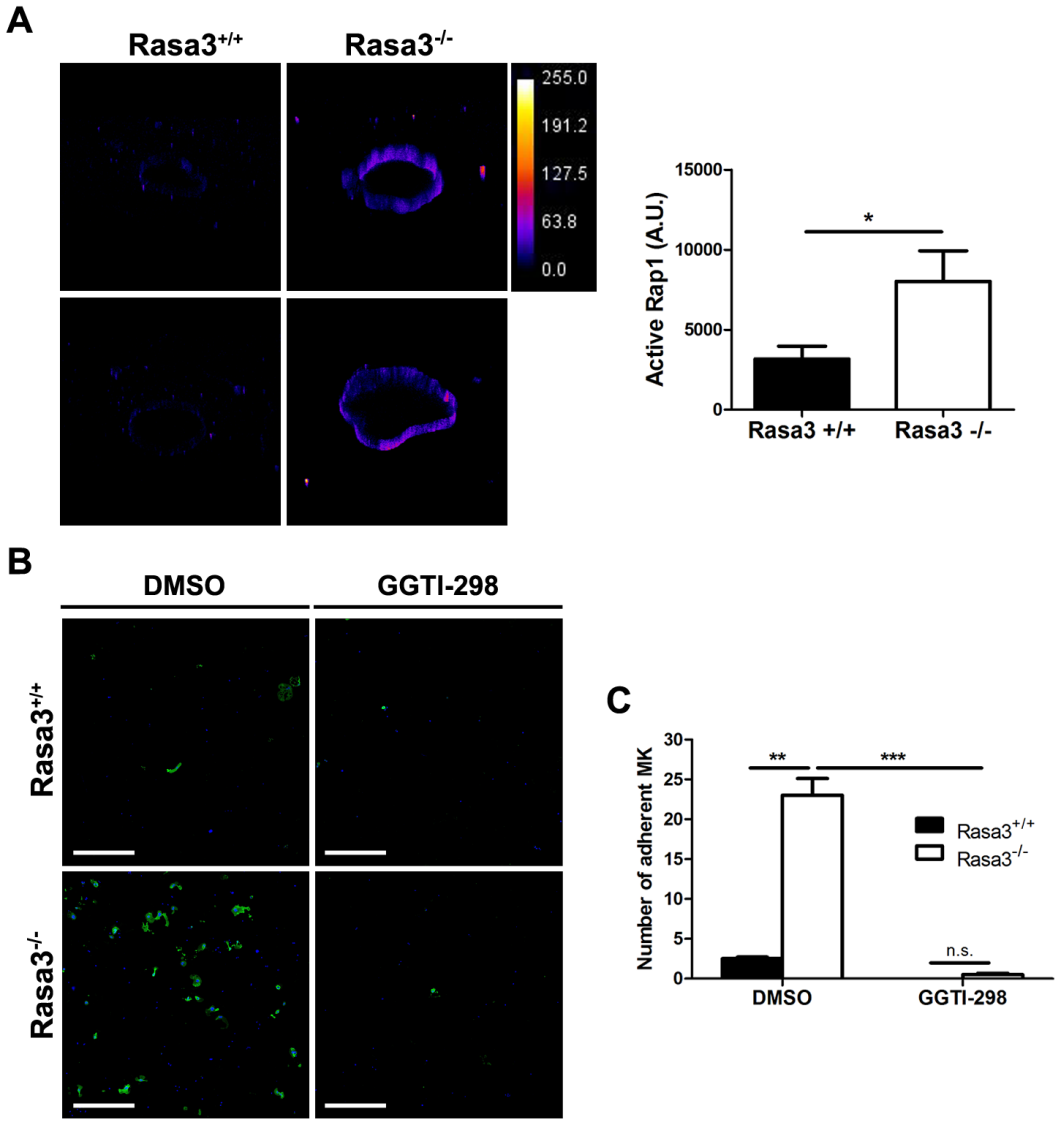
Increased active Rap1 in Rasa3^−/−^ megakaryocytes. Fetal liver cells (FLC) were isolated from E12.5 Rasa3^+/+^ and Rasa3^−/−^ embryos and cultured with TPO. **A.** Non adherent megakaryocytes on day 6 were analyzed for active, GTP-bound Rap1 by immunofluorescence using GST-RalGDS-RBD and a FITC-conjugated mAb against GST. 3D reconstruction of Rasa3^+/+^ (left panels) and Rasa3^−/−^ (right panels) megakaryocytes, along with the pseudocolor fluorescence intensity scale. The graph represents the intensity of active Rap1 staining, expressed in arbitrary units (A. U.), in Rasa3^+/+^ and Rasa3^−/−^ megakaryocytes. Two independent experiments (quantification of 50 megakaryocytes per experiment,) with mean ± SEM are presented. Statistics (unpaired *t* test): *: P<0.05 **B. and C.** Addition of the Rap1 GGTI-298 inhibitor to the culture medium (3 µM) abolished the abnormal adherent phenotype of Rasa3^−/−^ megakaryocytes. Fetal liver cell (FLC) were isolated from E12.5 Rasa3^+/+^ and Rasa3^−/−^ embryos and cultured with TPO for 3 days. Megakaryocytes were enriched on a BSA-gradient and incubated for 18 hours on Poly-D-Lysine- (PDL) coated plates in medium containing 10% FBS, in the presence or absence of GGTI-298. **B.** Representative confocal image of Rasa3^+/+^ and Rasa3^−/−^ PDL-adherent megakaryocytes after staining with CD41-APC (green) and DAPI (blue). Scale bar: 500 µm. **C.** The number of Rasa3^+/+^ and Rasa3^−/−^ adherent megakaryocytes was significantly decreased in GGTI-298 treated cells. Graph represents 2 independent experiments (mean ± SEM). Statistics (unpaired *t* test): **: P<0.01; ***: P<0.001.

Since the small GTPase Rap1, a Rasa3 substrate, controls inside-out and outside-in integrin signaling in megakaryocytes and platelets [25]–, the level of active, GTP-bound Rap1 was analyzed in mature Rasa3^+/+^ and Rasa3^−/−^ megakaryocytes. A significant 2 fold-increase in active Rap1 was observed in Rasa3^−/−^ megakaryocytes, as compared with Rasa3^+/+^ megakaryocytes, providing a molecular mechanism for the link between Rasa3, talin and integrin activation (Fig. 6a; P = 0.019). By contrast, no significant difference was detected in the level of active GTP-bound Ras between Rasa3^+/+^ and Rasa3^−/−^ megakaryocytes (GTP-Ras mean fluorescence intensity: Rasa3^+/+^ megakaryocytes: 670.1±117.9 arbitrary units (A. U.); Rasa3^−/−^ megakaryocytes: 706.2±56.7 A. U.; P = 0.48; 3 independent experiments, 50 megakaryocytes analyzed per field, 2 fields per FLC culture). In order to confirm the important role of Rap1 in the abnormal adherent phenotype of Rasa3^−/−^ megakaryocyte, outside-in experiments were performed in the presence of the Rap1 inhibitor GGTI-298, which is not active on Ras. Addition of GGTI-298 to the culture medium completely abolished the abnormal adhesion phenotype of Rasa3^−/−^ megakaryocyte, but had no effect on the proplatelet phenotype (Fig. 6b and 6c, and data not shown).

Altogether, these results indicate that the absence of Rasa3 increases Rap1 activation, and that Rap1 rather than Ras is probably responsible for the abnormal Rasa3^−/−^ adherent megakaryocyte phenotype. Thus, increased Rap1 activation in the absence of Rasa3 leads to constitutive activation of integrins and increased outside-in signaling.

### Altered platelet adhesion and activation in Rasa3^+/−^ mice

Platelet adhesion and activation were analyzed on platelets isolated from adult Rasa3^+/+^ and Rasa3^+/−^ mice (Fig. S4). Rasa3^+/−^ platelets adhesion to BSA-coated plates was significantly increased compared with Rasa3^+/+^ platelets (Fig. S4a). On fibrinogen-coated plates, a trend for an increase adhesion was detected in Rasa3^+/−^ platelets, but the difference with Rasa3^+/+^ platelets did not reach statistical significance (platelet counts per field of view (FOV) (means ± SEM); Rasa3^+/+^ platelets: 71±18 platelets/FOV; Rasa3^+/−^ platelets: 114±39 platelets/FOV; P = 0.12). In resting condition, two platelet activation markers were found altered in Rasa3^+/−^ platelets: the JON/A antibody binding to Rasa3^+/−^ platelets and the percentage of CD62P P-selectin positive Rasa3^+/−^ platelets were significantly increased, as compared with Rasa3^+/+^ platelets (Fig. S4b and S4c). No difference in the percentage of CD62P^+^ platelets was detected after stimulation with ADP or CRP (Fig. S4c). In resting condition, we found no difference in CD61 expression on Rasa^+/+^ and Rasa3^+/−^ platelets, whereas CD41 expression was significantly reduced on Rasa^+/−^ platelets; this data indicates that the increased JON/A binding to Rasa3^+/−^ platelets is not simply a consequence of an increased αIIβ3 integrin expression (Fig. S4d). Finally, platelet aggregation after ADP stimulation was similar in Rasa3^+/+^ and Rasa3^+/−^ platelets (Fig. S4e).

Altogether, these results indicate that Rasa3^+/−^ platelets present adhesion and activation defects in resting conditions, suggesting that a similar pathological mechanism is present both in megakaryocytes and platelets.

### Preleukemia in 4/24 SCID-Rasa3^−/−^ mice

**Figure 7:**
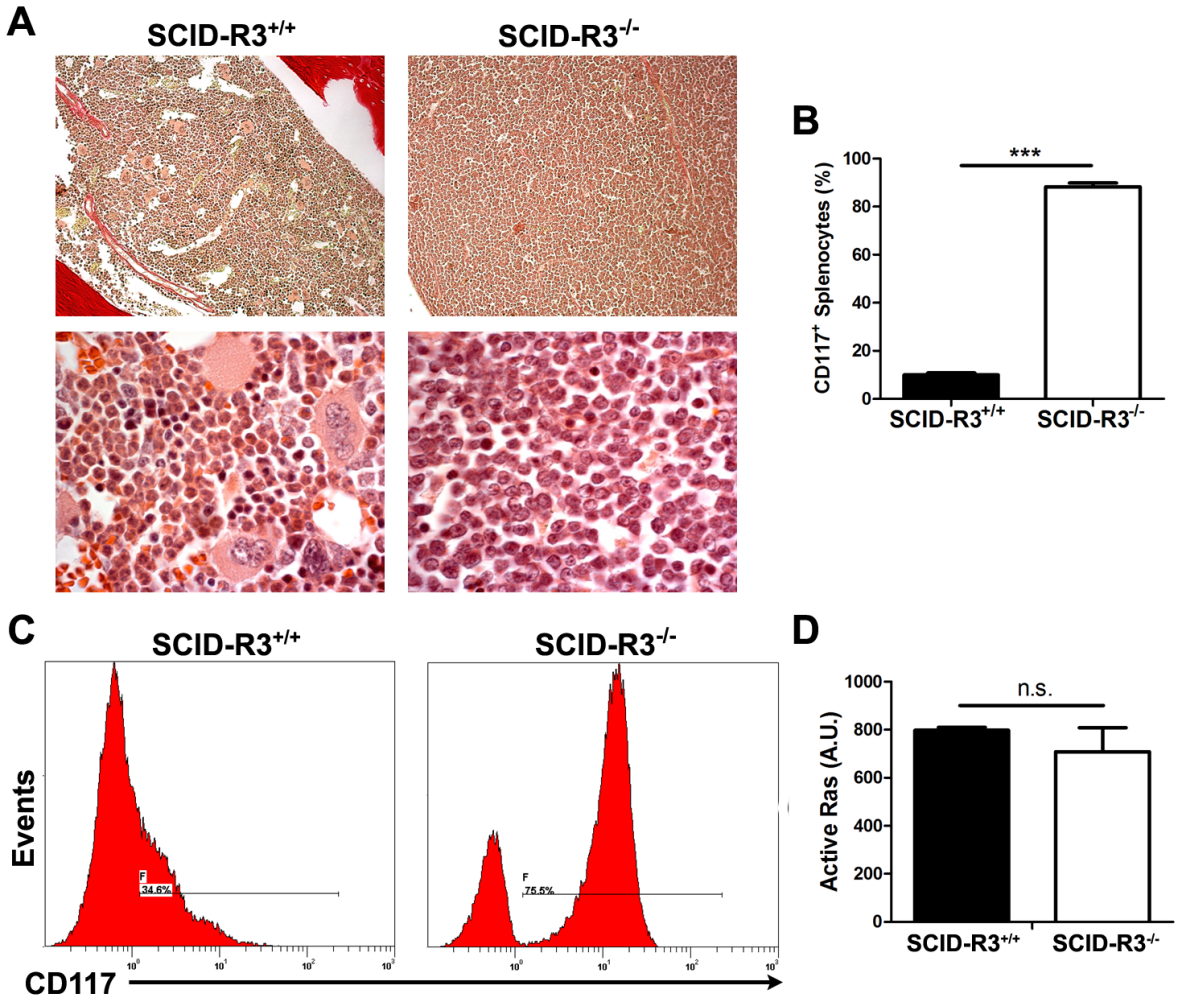
SCID-Rasa3^−/−^ mice develop a CD117^+^ CD38^+^ Sca-1^+^ cell preleukemia. A. Representative images of a hematoxylin/eosin-stained section of a femur from a SCID-Rasa3^+/+^ mouse and one of the four SCID-Rasa3^−/−^ mice with a homogeneous cellular infiltration of the bone marrow and the spleen (upper panels: magnification: ×20; lower panels: magnification: ×100). **B**. CD117^+^ splenocyte percentages in SCID-Rasa3^+/+^ (n = 10) and in the four SCID-Rasa3^−/−^ mice with a preleukemia. Statistics (unpaired *t* test): ***: P<0.001. **C**. Representative flow cytometry analysis of bone marrow cells from a SCID-Rasa3^+/+^ mouse (left histogram) and one of the four SCID-Rasa3^−/−^ (right histogram) mice with a preleukemia, using a CD117 antibody. The histograms show the CD117 fluorescence intensity and the relative number of cells (events). **D**. Fetal liver cells from Rasa3^+/+^ and Rasa3^−/−^ E12.5 embryos were stained with a CD117 antibody and analyzed for active GTP-bound Ras level by immunofluorescence using GST-Raf1-RBD and a FITC-conjugated mAb against GST. The graph represents the intensity of active GTP-bound Ras staining, expressed in arbitrary units (A. U.), in Rasa3^+/+^ and Rasa3^−/−^ CD117^+^ HSC. Mean ± SEM are presented.

In the ∼20% (4/24) remaining SCID-Rasa3^−/−^ mice, a very different phenotype was observed: a massive and homogeneous cellular infiltration was detected in the bone marrow and spleen, suggestive of a leukemia (Fig. 7a and data not shown). Adult naïve SCID mice intraperitoneally injected with 10^7^ splenocytes isolated from these SCID-Rasa3^−/−^ mice did not develop a similar proliferative disorder within 4 months after injection, suggesting the presence of a preleukemia rather than a leukemia in these 4 SCID-Rasa3^−/−^ mice (data not shown). No fibrosis was detected in the bone marrow of these 4 SCID-Rasa3^−/−^ mice. Flow cytometry analysis with a panel of antibodies revealed that cells massively infiltrating the bone marrow and the spleen were positive for CD117/c-Kit, CD38 and Sca-1, and negative for all other cell surface markers tested, including B220, CD3, MAR-1, Gr1, Mac1, Ter119, CD71, CD4, CD34 and F4.80 (Fig. 7b, 7c and data not shown). As expected, the percentage of B220^+^, CD3^+^, Gr1^int^ Mac1^+^, Ter119^+^ CD71^+^, CD41^+^ and F4.80^+^ cells was significantly decreased in the bone marrow and the spleen of these 4 mice (data not shown). These 4 mice had a reduced survival (survival range: 6–11 months after SCID mice irradiation/reconstitution) and a splenomegaly (spleen weight range: 0.185–1.062 g).

Collectively, these results indicate that about 20% of SCID-Rasa3^−/−^ mice develops a preleukemia with a massive infiltration of bone marrow and spleen with CD117^+^ Sca-1^+^ CD38^+^ cells, probably leading to bone marrow failure and premature death. They also suggest that Rasa3 is a potential tumor suppressor gene, acting may be on Ras, as proposed by Blanc et al. [18]. However, the level of active, GTP-bound Ras was similar in CD117^+^/c-Kit^+^ hematopoietic stem cells derived from Rasa3^+/+^ and Rasa3^−/−^ FLC cultures (Fig. 7d).

## Discussion

Using a Rasa3 catalytic mutant in FLC and irradiated/reconstituted SCID models, we show here that Rasa3 catalytic activity controls megakaryocyte development and differentiation into proplatelet forming megakaryocytes. In the irradiated/reconstituted SCID model, these megakaryocyte alterations are associated with thrombocytopenia, bleeding, regenerative anemia and decreased survival, as well as with bone marrow fibrosis, extramedular hematopoiesis and splenomegaly.

An increased percentage of mature megakaryocytes with an abnormal morphology was detected in bone marrow cells from irradiated/reconstituted SCID mice when Rasa3 catalytic activity was inactivated. This increased percentage was associated with a slightly decreased percentage of progenitors with megakaryocyte potential, suggestive of a megakaryopoisis alteration. An obvious megakaryopoiesis alteration was also detected in Rasa3^−/−^ FLC culture, where the number of CFU for immature megakaryocyte was significantly decreased and associated with the presence of numerous mature megakaryocytes. Ploidy in these Rasa3^−/−^ abnormal megakaryocytes was also slightly altered. On the contrary to active Ras level, level of active GTP-bound Rap1 was significantly increased in Rasa3^−/−^ megakaryocytes. Interestingly, the small GTPase Rap1 is both a Rasa3 substrate and a well known regulator of integrin signaling in megakaryocytes and platelets [2], [25]–[29]. Both inside-out and outside-in integrin signaling are controlled by Rap1, including αIIbβ3 signaling. Thus, the increased active GTP-bound Rap1 level detected in Rasa3^−/−^ megakaryocytes represents a plausible molecular mechanism linking Rasa3 to integrin signaling and the altered megakaryocyte development and differentiation. Indeed, altered inside-out and outside-in integrin signaling in Rasa3^−/−^ megakaryocytes probably results in the adherence and motility defects that we observed in this study. These defects may secondarily lead to an abnormal distribution of megakaryocytes between osteoblastic and vascular niches and to altered megakaryopoiesis. Constitutive activation of integrin signaling in Rasa3^−/−^ megakaryocytes is associated with alteration in actin cytoskeleton organization, including a lack of stress fiber assembly, in talin recruitment to the plasma membrane and in cell adherence and spreading that occurred independently of integrin ligands. These alterations probably prevent terminal differentiation of Rasa3^−/−^ megakaryocytes since megakaryocyte αIIbβ3 and β1 integrins are known to control proplatelet production and platelet release [30]–[32]. Moreover, stress fiber assembly is known to require optimal β1 integrin activation, a process also regulated by αIIbβ3 integrin [33], [34]. In future work, it will be important to analyze Rasa3^−/−^ platelets, since integrins play also important roles in these cells. Our preliminary studies indicate that unstimulated Rasa3^+/−^ platelets have altered adhesion to BSA-coated plates and activation, as compared with Rasa3^+/+^ platelets, thus mimicking defect of Rasa3^−/−^ megakaryocytes.

Interestingly, constitutive αIIbβ3 integrin activation in human megakaryocytes mimics most of the Rasa3^−/−^ megakaryocyte phenotypical traits. Indeed, in rare thrombocytopenic patients with activating mutations in ITGA2B or ITGB3 genes, megakaryocyte spreading on fibrinogen is abnormal, with 50% of spread cells showing a disordered actin distribution where focal adhesion points are more evident than stress fibers [21]. Sustained and substrate-independent activation of the outside-in αIIbβ3 signaling was detected in megakaryocytes of these patients, leading to severely impaired proplatelet formation and congenital thrombocytopenia [20]–[22]. It is noteworthy here that these patients do not develop the entire Rasa3^−/−^ phenotype, like megakaryocytosis and bone marrow fibrosis, and its consequences. This discrepancy suggests that Rasa3 has additional function beside the control of integrin signaling, and/or that the enzyme has slightly different roles in man and mouse.

In a recent report, Peters and collaborators have described a new spontaneous mutant mouse with a missense mutation in the Rasa3 protein [18]. The G125V Rasa3^Scat^ mutation causes mislocalization of the protein to the cytosol and phenotypical traits that are clinically and biologically most often different from SCID-Rasa3^−/−^ and Rasa3^−/−^ phenotypes (Table S5). Indeed, Rasa3^Scat/Scat^ mice have a cyclic phenotype of crisis-remission with a first embryonic to P9 wave of lethality – which affect ∼60% of the mutant mice – followed by a second wave of lethality at P30 (affecting 94% of the first crisis survivors). This unexplained cyclic phenotype is fully transferable via hematopoietic stem cells injection into SCID or RAG^−/−^ mice, ruling out the possibility that expression of the mutant Rasa3^Scat^ protein outside the hematopoietic system is responsible for the different phenotype [18]. Another notable difference between Rasa3^Scat/Scat^ and SCID-Rasa3^−/−^ mice is the presence of a delayed erythropoiesis in the former mice. By contrast, in SCID-Rasa3^−/−^ mice, many hallmarks of regenerative anemia are present. It is noteworthy that no bone marrow fibrosis nor extramedullar hematopoiesis have been reported in the Rasa3^Scat/Scat^ model, and no mechanism was presented to explain the severe Rasa3^Scat/Scat^ thrombocytopenia. Finally, no evidence for predisposition to oncogenesis was observed in Rasa3^Scat/Scat^ mice, but the very small numbers of homozygous mice that survive the second crisis period (∼6% of Scat/Scat newborns) may explain this difference and preclude more extensive analysis. The cause of the major differences between the Rasa3^Scat/Scat^ and SCID-Rasa3^−/−^ phenotypes is currently not known, but may be due to the different mutation present in the Rasa3 protein - affecting protein localization and enzymatic activity, respectively - and/or to the different genetic background of the two models. Indeed, relocalization of the Rasa3^Scat/Scat^ protein from the membrane to the cytosol may eventually create a new function in this cell compartment and lead to phenotypic alterations that are not present in mice expressing a catalytically-inactive and truncated Rasa3 protein.

About 20% of SCID-Rasa3^−/−^ mice develop a preleukemia characterized by a massive infiltration of bone marrow and spleen with CD117^+^ Sca-1^+^ CD38^+^ cells, a phenotype very similar to acute myeloid leukemia in man. The exact mechanism of this preleukemia was not defined in this work, but active GTP-bound Ras level was similar in Rasa3^+/+^ and Rasa3^−/−^ fetal liver CD117^+^ hematopoietic stem cells. However, our studies in the human K562 leukemic cell line which overexpresses Rasa3 suggest that Rasa3 is a probable negative regulator of proliferation in these cells (Fig. S5). Alternatively, it has been reported that β1 and β3 integrin signaling regulates the balance among hematopoietic stem cell self-renewal, differentiation and quiescence in the osteoblastic niche [35], [36]. Furthermore, β1 and β3 integrins can regulate stem cell functions via direct or indirect participation in cellular signaling [37], providing a potential mechanism to explain the predisposition to preleukemia in a minor percentage of SCID-Rasa3^−/−^ mice.

In conclusion, our results demonstrate that mice with a catalytic inactivation of Rasa3 protein in the hematopoietic system develop a lethal syndrome characterized by defects during megakaryocyte development and differentiation, and leading to a severe thrombocytopenia. This syndrome is associated with Rap1 and integrin signaling alterations and a predisposition to develop preleukemia.

## Materials and Methods

### Ethics statement

All animal studies were authorized by the Animal Care Use and Review Committee of the Université de Liège and of the Université Libre de Bruxelles.

### Mice

Rasa3^−/−^ mice with Rasa3 exons 11 and 12 replaced by a neomycin resistance cassette express a catalytically-inactive Rasa3 truncated protein [16]. These mice were analyzed on a hybrid 129/SvJ×C57BL/6J genetic background. C.B.-17 SCID mice were purchased from Charles River, Belgium. All mice were bred in a specific pathogen free facility at the GIGA–Research Centre. The Rasa3 genotype was determined by PCR as previously described [16]. For reconstitution, 4–6 week-old C.B.-17 SCID mice were irradiated (200 rad) and a total homogenate of E12.5 fetal liver cells (FLC) obtained from Rasa3 embryos was intravenously injected. SCID-Rasa3^−/−^ mice were killed and analyzed either when moribund (ie presenting a severely reduced mobility and/or feeding incompatible with a more than 2 days survival) or 14 months after irradiation/reconstitution.

### Fetal liver cells (FLC) isolation and megakaryocyte differentiation

Individual liver was recovered from E12.5 embryo and single cell suspension was prepared by passage through a 23-gauge needle. Recovered cells were cultured in DMEM (Gibco) supplemented with 10% heat-inactivated FBS, 2 mM L-Glutamine, 50 U/mL Penicillin, 50 ng/mL streptomycin, 0.1 mM nonessential amino acids and 50 ng/ml of recombinant mouse TPO for megakaryocyte differentiation (PreProtech).

### Bone marrow explants analysis

Bone marrow from SCID-Rasa3^+/+^ and SCID-Rasa3^−/−^ femurs were flushed with PBS. The marrow was cut in 1 mm transverse sections and placed in an incubation chamber containing complete DMEM medium. Chamber was maintained at 37°C for 6 h. Megakaryocytes at the periphery of the explant were observed under a confocal microscope (Nikon A1R, 20× objective). Each experiment was performed in duplicates. One transversal section was used to determine by flow cytometry the number of CD41^+^ cells present in the explant. Images were acquired sequentially at 10 min intervals and processed with NIS-software and ImageJ. Three mice from each genotype were analyzed.

### Inside-out αIIbβ3 integrin and outside-in integrins signaling in megakaryocytes

FLC from Rasa3^+/+^ and Rasa3^−/−^ embryos were cultured in the presence of TPO as described above. On day 3, recovered cells were enriched for mature megakaryocytes on a 1.5–3% bovine serum albumin (BSA) gradient under gravity for 45 min at room temperature. The percentage of mature megakaryoctes in the enriched population was always over 70%. Cells were resuspended in Tyrode's buffer containing 1 mM CaCl_2_ and 1 mM MgCl_2_ for 3 h. For inside-out integrin signaling, cells were incubated for 30 min at room temperature with FITC-fibrinogen (250 µg/ml) and 100 ng/ml TPO, 1 mM MnCl_2_ or nothing, in the presence or absence of 10 mM EDTA. After a 10-fold dilution with PBS containing 1 µg/ml propidium iodide, fibrinogen binding was quantified by flow cytometry [22]. Specific fibrinogen binding was defined as binding that was inhibited by 10 mM EDTA. To compare independent experiments, specific fibrinogen binding was expressed as a percent of maximal binding obtained in the presence of 1 mM MnCl_2_, an activator of integrins. For outside-in integrin signaling, coverslides were coated with murine fibrinogen (100 µg/ml), collagen-I (35 µg/ml) or Poly-D-Lysine (PDL, 15 µg/ml) for 1 h at room temperature, blocked with denatured BSA (5 mg/ml) for 30 min and washed with PBS before use. Cells (25×10^3^) were incubated for 18 h on the indicated substrate and non adherent cells were removed. Adherent cells were fixed in 10% formalin, permeabilized with 0.2% Triton X-100 in PBS and stained as described below. Cells were then analyzed by confocal microscopy and ImageJ Software. For Rap1 inhibitor studies, purified mature megakaryocytes were cultured over PDL coated-plates as in outside-in experiments in the presence of 3 µM GGTI-298 (Sigma) or DMSO as control. Adherent cells were fixed in 10% formalin, permeabilized with 0.2% Triton X-100 in PBS and stained as described above. Cells were then analyzed by confocal microscopy and ImageJ Software.

### Flow cytometry analysis and antibodies

A single-cell suspension of femur bone marrow was prepared by flushing the bones with PBS followed by gentle disaggregation through Pasteur pipette. Cells were released from spleen by gentle disruption with a piston of syringe. Spleen cells were treated with ACK buffer to lyse erythrocytes and washed once with PBS. Cells were incubated with 2.4G2 to saturate Fcγ receptors II and IIIa before staining with primary and secondary antibodies in PBS containing 0.1% FBS and 0.1% NaF for 20 min, and washed with the same solution before flow cytometric analysis on a FC 500 (Beckman Coulter). Cell counts were determined by adding fluorospheres (Flow-Count Fluorospheres, Beckman Coulter) to the cell suspension, as described by the manufacturer. The following anti-mouse biotinylated or fluorochrome-conjugated antibodies were obtained from BD Pharmingen: anti-CD3ε, anti-CD71, anti-CD41 and anti-CD117. Anti-B220, anti-F4/80, anti-IgM, anti-Mac1, anti-Sca-1, anti-CD34, anti-CD38, anti-Ter119, anti-CD41 and anti-Gr1, as well as streptavidine-cychrome 5 were obtained from eBioscience. Anti-FcεRIa (Mar-1) was obtained from O. Leo's laboratory (Université Libre de Bruxelles, Belgium). JON/A antibody was obtained from Emfred Analytics. Fetal liver cell were analyzed on a FACS CantoII (Beckman Coulter). For hematopoietic stem and megakaryocyte progenitor cells staining, anti-mouse biotinylated or fluorochrome-conjugated antibodies specific for Ter-119, Gr1, Mac1, CD4, CD8, CD5, IL7Rα, B220 and c-Kit (CD117) were used to define the c-Kit^+^ Lin^−^ cell population [19], [38]. Then, anti-Sca-1, anti-CD34 and anti-Flk2/Flt3 were used to define the hematopoietic stem cells, whereas anti-Sca-1, anti-FcRγII/III and anti-CD150 were used to define the megakaryocytes progenitor cells (all antibodies were from eBioscience, except anti-Flk2, from BD Pharmingen and anti-CD150, from BioLegend). Streptavidin phycoerythrin-Texas Red was from Invitrogen. Debris, aggregates and propidium iodide-positive dead cells were first excluded. Cells were analyzed using an LSRII flow cytometer (Becton Dickinson). Data were analyzed with FlowJosoftware (Tree Star, Ashland, OR).

### Ploidy assay

Fetal liver cells were stained for CD41 as described above and fixed with 5% formalin for 15 min. Cells were permeabilized in PBS containing 0.25% Tx-100 for 5 min at 4°C. DNA was stained with DAPI for 20 min and DNA content in CD41^+^ cells was determined by flow cytometry.

### Histology

Spleen and liver were fixed in paraformaldehyde 4% and embedded in paraffin following standard procedures. Femurs were fixed in paraformaldehyde 3.7%, decalcified in 0.5M EDTA pH 8 for one week and then processed as spleen and liver. Serially cut 5-µm-thick sections were stained with hematoxylin/eosin or Sirius Red (for femur) according to standard protocols.

### Immunohistochemistry of spleen and femur

Spleen was processed as described and sections were stained with an anti-B220 antibody [39]. Femur sections were stained with a rabbit polyclonal anti-von Willebrand Factor (vWF) antibody from Dako. For quantification of megakaryocytes in osteoblastic and vascular niches, the whole diaphysis of three consecutive femur sections was scanned with a conventional microscope (20× objective) for vWF^+^ cells, as described [40]. Megakaryocytes in the osteoblastic niche were calculated as the number of megakaryocytes in contact with the endosteal border. Megakaryocytes in the vascular niche were calculated as the number of megakaryocyte per vessel border. Osteoblastic and vascular borders were calculated with ImageJ software. Results are means ± SEM of 3 mice per genotype.

### Blood analysis

Platelet counts were determined with Unopette (Becton Dickinson). Red cells, total white cells, lymphocytes, neutrophils, eosinophils, basophils, hemoglobin, hematocrit and red cell volume were quantified with a Cell Dyn 3500 analyzer (Abott Diagnostic). Serum erythropoietin and thrombopoietin levels were determined with ELISA mouse EPO and mouse TPO Quantikine kits (R&D Systems). Blood smears were stained with Giemsa's, methylene blue and Romanowsky's solutions.

### Immunofluorescence and confocal microscope analysis

Immunofluorescence studies using conventional and confocal microscopes were performed on total FLC cultured in the presence of TPO, on purified mature megakaryocytes and on FL hematopoietic stem cells. Cells were fixed in 5% formalin for 15 min, washed, permeabilized with 0.2% Tx-100 in PBS containing 2% of FBS for 15 min and incubated 1 h at room temperature with APC-conjugated anti-CD41 (MW Reg30, eBioscience) for megakaryocyte or CD117 (BD Pharmingen) for HSC. Active, GTP-bound Rap1 or Ras immunofluorescence was detected using GST-RalGDS-RBD or GST-Raf1-RBD, respectively, and a FITC-conjugated mAb against GST (Santa Cruz) as described [41] Negative controls included the omission of GST-RalGDS-RBD/GST-Raf1-RBD, the substitution of GST-RalGDS-RBD/GST-Raf1-RBD with GST and the substitution of the anti-GST antibody with an irrelevant FITC-conjugated mouse IgG. After several washes, phalloidin-TRICT (Sigma) and DAPI (Sigma) were added for 20 min in PBS. After 3 washes in PBS, samples were mounted in ProLong (Invitrogen) for observation under a confocal microscope (NikonA1R) and/or an epifluorescence microscope (Nikon Eclipse 90i). For active Rap1 or Ras images, z-sections of 0.150 microns were acquired from megakaryocytes or HSC. Pseudocolor scale was used to depicture the intensity of active Rap1 or Ras staining along the cell membrane. ImageJ was used to quantify the intensity of active Rap1 or Ras staining on each cell. All images were acquired and analyzed in the same conditions.

For immunofluorescence studies of adherent megakaryocyte, cells were fixed with 10% formalin for 15 min, washed, permeabilized with 0.2% Tx-100 in PBS containing 2% of FBS for 15 min and incubated 1 h at room temperature with the indicated primary and secondary antibodies. After several washes, phalloidin-TRICT (Sigma) and DAPI (Sigma) were added for 20 min in PBS. After 3 washes in PBS, samples were mounted in ProLong (Invitrogen) for observation under a confocal microscope (NikonA1R). The following antibodies were used: APC-conjugated anti-CD41 (MW Reg30, eBioscience), anti-Rap1 (Millipore), anti-Talin-FITC and anti-rabbit-alexa 488.

### CFU-Mk assay

A collagen-based system (MegaCult-C, StemCell Technologies, Inc.) was used for the colony assay. Briefly, 1.25×10^5^ freshly isolated fetal liver cells were resuspended in IMDM completed with recombinant mouse TPO (50 ng/ml), IL-3 (20 ng/ml) and IL-6 (10 ng/ml), followed by addition of cold collagen. Suspension was dispensed into 2 wells of a four chamber slide (Millipore) for duplicates. Cultures were kept at 37°C in a 5% CO2 atmosphere for 3 days. The collagen matrix was then fixed in a methanol–acetone solution (1∶3), at room temperature for 20 min for colony fixation. Slides were then allowed to air dry for 15 min and stained for Acetylcholinesterase. For scoring, acetylcholinesterase-positive colonies with 3 or more immature megakaryocytes of about 10 µm of diameter were scored as CFU-Mk. Mature megakaryocytes averaged approximately 30 µm in diameter.

### Preparation of mouse washed platelets

Eight- to twelve-week old male mice were bled under sodium pentobarbital anesthesia from the retro-orbital plexus. Blood was collected on acid citrate dextrose (ACD: 93 mM Na_3_-citrate, 7 mM citric acid, 14 mM dextrose, pH 6.0) containing 1 U/ml apyrase (Grade I, Sigma) in a volume ratio of ACD to blood of 1∶6. Blood was centrifuged for 5 s at 800× *g* followed by 5 min at 100× *g* to obtain platelet rich plasma (PRP). PRP was diluted 3 fold in ACD containing 1 U/ml apyrase and centrifuged at 1000× *g*. The platelet pellet was resuspended at a concentration of 3×10^8^/ml in Tyrode's buffer (137 mM NaCl, 12 mM NaHCO_3_, 2 mM KCl, 0,34 mM Na_2_HPO_4_, 1 mM MgCl_2_, 5,5 mM glucose, 5 mM Hepes, 0.35% BSA).

### Platelet adherence assay

In order to test the adhesion of unstimulated platelets to BSA-coated surface, 3.5×10^6^ platelets in 300 µl of tyrode's buffer were added to each well of a 8 chambers slide (Millipore) and incubated for 45 min in a CO_2_ incubator at 37°C. Adherent platelets were washed twice with PBS, fixed with 10% formalin, and stained with phalloidin-TRICT.

### Flow cytometry analyses of platelet activation

Washed platelets were stimulated or not with ADP (25 µM) or collagen-related peptide (CRP) (1 µg/ml),under non-stirring conditions. After 15 minutes of activation, saturating concentrations of FITC-conjugated CD62 anti-P-selectin and PE-conjugated JON/A antibodies were added to the platelets, and incubations were continued for additional 15 minutes in the dark. Samples were fixed before the analysis with a FACS Calibur flow cytometer (BD Biosciences).

### Platelet aggregation analysis

Light transmission was recorded during platelet aggregation induced by ADP (50 µM) in the presence of 2 mM CaCl_2_ on a Chrono-Log Lumi-Aggregometer (Havertown, PA).

### Proliferation assay on a Rasa3-inducible K562 leukemic cell line

The Rasa3-tet-ON-inducible K562 cell line was generated by GEnTarget Inc. Briefly, Rasa3 expression and TetR repressor lentiviruses were generated and cotransduced in K562 cell by the company. K562 mutant cell line (K562-Rasa3) was cultured in IMDM supplemented with 10% heat-inactivated FBS, 2 mM L-Glutamine, 50 U/mL Penicillin, 50 ng/mL streptomycin, 0.1 mM nonessential amino acids, 10 µg/ml blasticidin and 1 µg/ml puromycin. Treatment of K562-Rasa3 cells with tetracycline (2 µg/ml) induced Rasa3 expression from the lentiviral constructs after 48 h. For the proliferation assay, 4×10^5^ cells per ml were cultured in the absence or presence of tetracycline for 12 days. At the indicated days, number of alive cells was counted with a hemocytometer. Death cells were excluded by trypan blue staining. Rasa3 expression was confirmed by western blot. Two independent experiments were performed in duplicates-triplicates.

### Statistics

Results are expressed as means ± SEM. Statistical analyses were performed with Graphpad Prism 3.0. The test used for each experiment is described in the corresponding legend. For each test, a difference of P<0.05 was considered significant.

## Supporting Information

Figure S1 Bone marrow histology of SCID-Rasa3^+/+^ and SCID-Rasa3^−/−^ mice. (**A**) Sirius Red-stained sections of age-matched SCID-Rasa3^+/+^ and moribund SCID-Rasa3^−/−^ femurs. Numerous collagen trabeculae are detected in the cavity of the mutant femur, while the cavity of SCID-Rasa3^+/+^ femur was free of collagen trabeculae. (**B**) Hematoxylin/eosin-stained sections of femur isolated from age-matched SCID-Rasa3^+/+^ and moribund SCID-Rasa3^−/−^ mice. Asterisks indicate megakaryocytes. A similar cell density is observed in the cavity of SCID-Rasa3^+/+^ and SCID-Rasa3^−/−^ femurs. Scale bars: 50 µm.
(TIF)

Figure S2 Abnormal splenic architecture and liver hematopoiesis in SCID-Rasa3^−/−^ mice. Sections of age-matched SCID-Rasa3^+/+^ (left) and moribund SCID-Rasa3^−/−^ (right) spleen were stained with (**A**) hematoxylin/eosin (H/E) or (**B**) a B220 antibody (B220). In SCID-Rasa3^−/−^ spleen, the limits between red and white pulps are ill defined and the red pulp is infiltrated by cells of various sizes; the B cell compartment is also disorganized. **C.** Sections of age-matched SCID-Rasa3^+/+^ (left) and moribund SCID-Rasa3^−/−^ (right) liver were stained with hematoxylin/eosin (H/E). Liver hematopoiesis (arrow) is observed in SCID-Rasa3^−/−^ mice, but never in SCID-Rasa3^+/+^ mice. Inset: same image at higher magnification.
(TIF)

Figure S3 Regenerative anemia in SCID-Rasa3^−/−^ mice. Blood analyses were performed on age-matched SCID-Rasa3^+/+^ and moribund SCID-Rasa3^−/−^ mice. Mean ± SEM of red cell concentration (**A**), hemoglobin concentration (**B**), hematocrit (**C**) and red cell volume (**D**) in SCID-Rasa3^+/+^ (black columns, n = 7) and SCID-Rasa3^−/−^ (white columns, n = 11) mice. Representative images of anisocytosis with polychromasia (**E**), of Howell-Jolly bodies (**F**), of increased reticulocytosis (**G**) and of metarubricytes (**H**) observed on blood smear from moribund SCID-Rasa3^−/−^ mice. These alterations were not observed in age-matched SCID-Rasa3^+/+^ mice. **I.** Mean ± SEM of erythropoietin concentrations in age-matched SCID-Rasa3^+/+^ (black column, n = 8) and moribund SCID-Rasa3^−/−^ (white column, n = 8) mice. Together, these alterations are classically associated with a regenerative anemia. Scale bars: 5 µm. Statistics (unpaired *t* test): *: P<0.05; **: P<0.01.
(TIF)

Figure S4 Altered platelet adherence and activation in adult Rasa3^+/−^ mice. Unstimulated platelets were isolated from 8 week-old Rasa3^+/+^ and Rasa3^+/−^ mice. **A.** After 45 min, an increased number of Rasa3^+/−^ platelets adhered to BSA-coated plates, as compared with Rasa3^+/+^ platelets. Mean ± SEM of platelet counts per field of view (FOV) from two independent experiments performed in duplicate are represented. Statistics (unpaired *t* test): **: P<0.01. Representative images of Rasa3^+/+^ and Rasa3^+/−^ adherent platelets after 45 min, stained with phalloidin-TRICT (actin, red). **B.** Mean ± SEM of the mean fluorescence intensity (MFI) of the JON/A antibody binding to the high affinity conformation of the integrin αIIbβ3 on Rasa3^+/+^ and Rasa3^+/−^ platelets in resting condition. Results are representative of three separate experiments. Statistics (unpaired *t* test): *: P<0.05. **C.** Mean ± SEM of the percentage of CD62P^+^ platelets in non stimulated condition (n. s.) and after ADP (25 µM) or CRP (1 µg/ml) stimulation. Results are representative of three separate experiments. Statistics (unpaired *t* test): *: P<0.05. **D.** Mean ± SEM of the mean fluorescence intensity of CD61 and CD41 expression on Rasa3^+/+^ and Rasa3^+/−^ platelets. Results are representative of three separate experiments. Statistics (unpaired *t* test): ***: P<0.001. **E.** Platelet aggregation assay revealed no aggregation defect in Rasa3^+/−^ platelets in response to ADP (50 µM), as compared with Rasa3^+/+^ platelets. Results are representative of three separate experiments.
(TIF)

Figure S5 Effect of Rasa3 expression on K562 leukemic cell proliferation. Rasa3 expression in a mutant K562 leukemic cell line was induced by adding tetracycline in the culture medium for 12 days. At days 7 and 12, Rasa3 expression was analyzed by western blot and the number of living cells was measured with a hemocytometer. Graph represents the number of cells in the culture at days 7 and 12 (mean ± SEM, 3 independent experiments, each performed in duplicates/triplicates). Statistics (One-way anova): * P<0.05.
(TIF)

Table S1 Total numbers of T and B cells were determined in the spleen of SCID-Rasa3^+/+^, SCID-Rasa3^+/−^ and SCID-Rasa3^−/−^ mice 6 weeks after irradiation/reconstitution by flow cytometry on the basis of 145-2C11 and B220 expression. A trend for higher B220^+^ B cell number was observed in SCID-Rasa3^−/−^ mice as compared with SCID-Rasa3^+/+^ mice, but the difference did not reach statistical significance (P = 0.053, unpaired *t* test). Red blood cell, blood platelet and bone marrow megakaryocyte counts as well as spleen weight were also analyzed 6 weeks after irradiation/reconstitution. No significant difference was observed between SCID-Rasa3^+/+^ and SCID-Rasa3^−/−^ mice. Megakaryocyte counts per field of view were obtained with a ×20 objective, 3 fields per mouse, 5 SCID-Rasa3^+/+^ and 4 SCID-Rasa3^−/−^ mice.
(DOC)

Table S2 Bone marrow cells were isolated from SCID-Rasa3^+/+^ and SCID-Rasa3^−/−^ mice 2 months after irradiation/reconstitution, incubated with antibodies directed against cell surface markers and analyzed by flow cytometry for the percentage of cells within the bone marrow cells or within a subpopulation of bone marrow cells defined by specific markers.
(DOC)

Table S3 Age-matched SCID-Rasa3^+/+^ and moribund SCID-Rasa3^−/−^ mice were analyzed for their total number of nucleated splenocytes and, after flow cytometry with relevant antibodies, for their percentages (%) and cell numbers (n) of splenic mature (macrophages, T and B cells) and immature (megakaryocytes, myeloid cells, hematopoietic progenitors and erythroblasts) cells. Results indicate that in SCID-Rasa3^−/−^ mice, total number of nucleated splenocytes as well as percentage and number of immature splenic cells are significantly increased, consistent with a markedly increased hematopoiesis in the spleen of these mice, as compared with SCID-Rasa3^+/+^ mice. By contrast, the percentage of mature cells is decreased in the spleen of SCID-Rasa3^−/−^ mice, as compared with SCID-Rasa3^+/+^ mice, although their number is increased, a probable consequence of the increased hematopoiesis in this organ.
(DOC)

Table S4 Age-matched SCID-Rasa3^+/+^ and moribund SCID-Rasa3^−/−^ mice were analyzed for their total number of white cells and circulating neutrophils, lymphocytes, monocytes and eosinophils on Giemsa-stained blood smears.
(DOC)

Table S5 Genetic, biological and phenotypical differences between Rasa3^Scat/Scat^, Rasa3^−/−^ and SCID-Rasa3^−/−^ mice.
(DOC)
