# Supplementary figures and images for: Rasa3 Controls Megakaryocyte Rap1 Activation, Integrin Signaling and Differentiation into Proplatelet
Source: PLoS Genet. 2014 Jun 26;10(6):e1004420. doi: 10.1371/journal.pgen.1004420 (PMC4072513; doi:10.1371/journal.pgen.1004420)

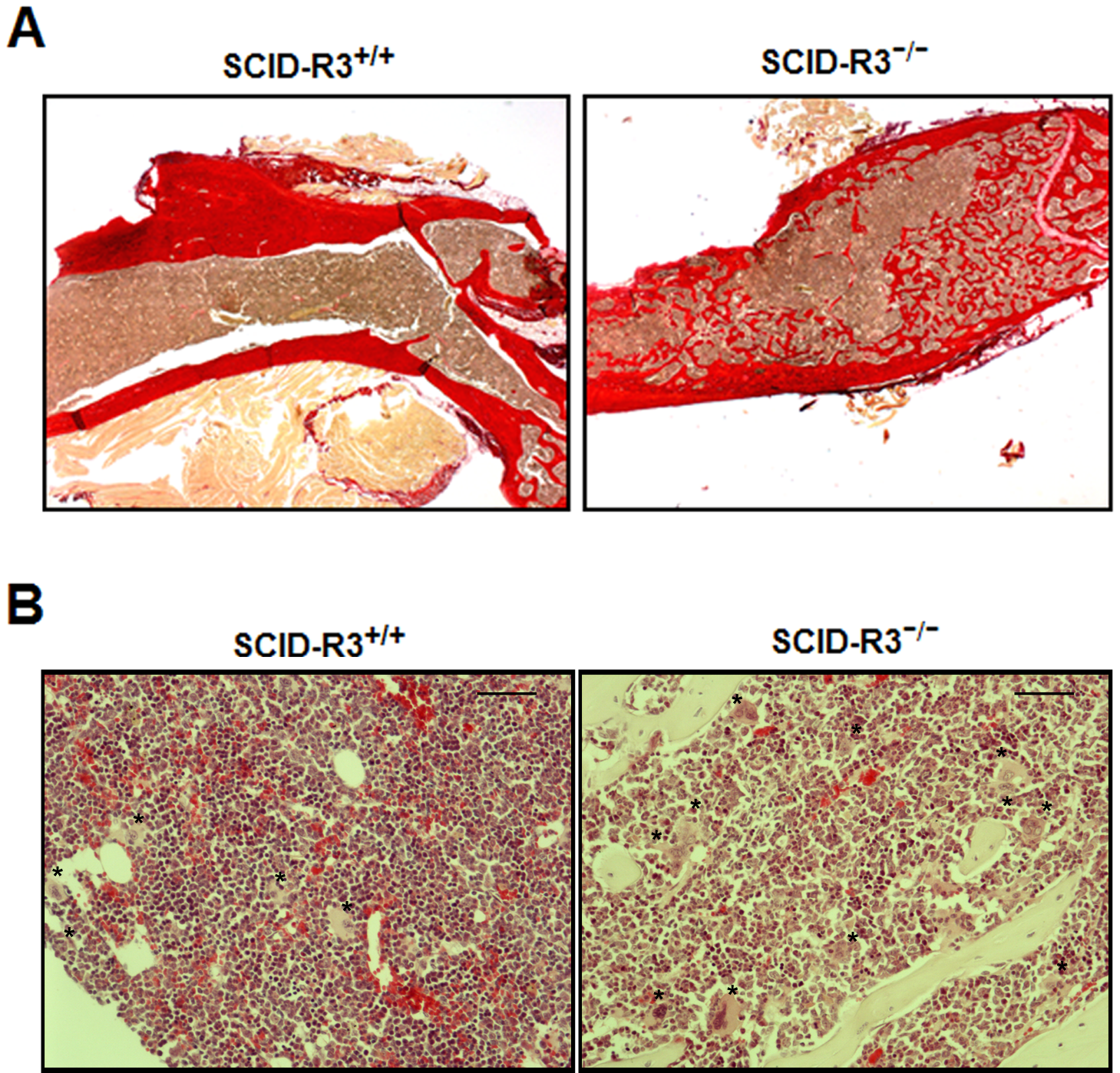

Supplement: Figure S1 — Bone marrow histology of SCID-Rasa3+/+ and SCID-Rasa3−/− mice. (A) Sirius Red-stained sections of age-matched SCID-Rasa3+/+ and moribund SCID-Rasa3−/− femurs. Numerous collagen trabeculae are detected in the cavity of the mutant femur, while the cavity of SCID-Rasa3+/+ femur was free of collagen trabeculae. (B) Hematoxylin/eosin-stained sections of femur isolated from age-matched SCID-Rasa3+/+ and moribund SCID-Rasa3−/− mice. Asterisks indicate megakaryocytes. A similar cell density is observed in the cavity of SCID-Rasa3+/+ and SCID-Rasa3−/− femurs. Scale bars: 50 µm. (TIF) [file pgen.1004420.s001.tif]

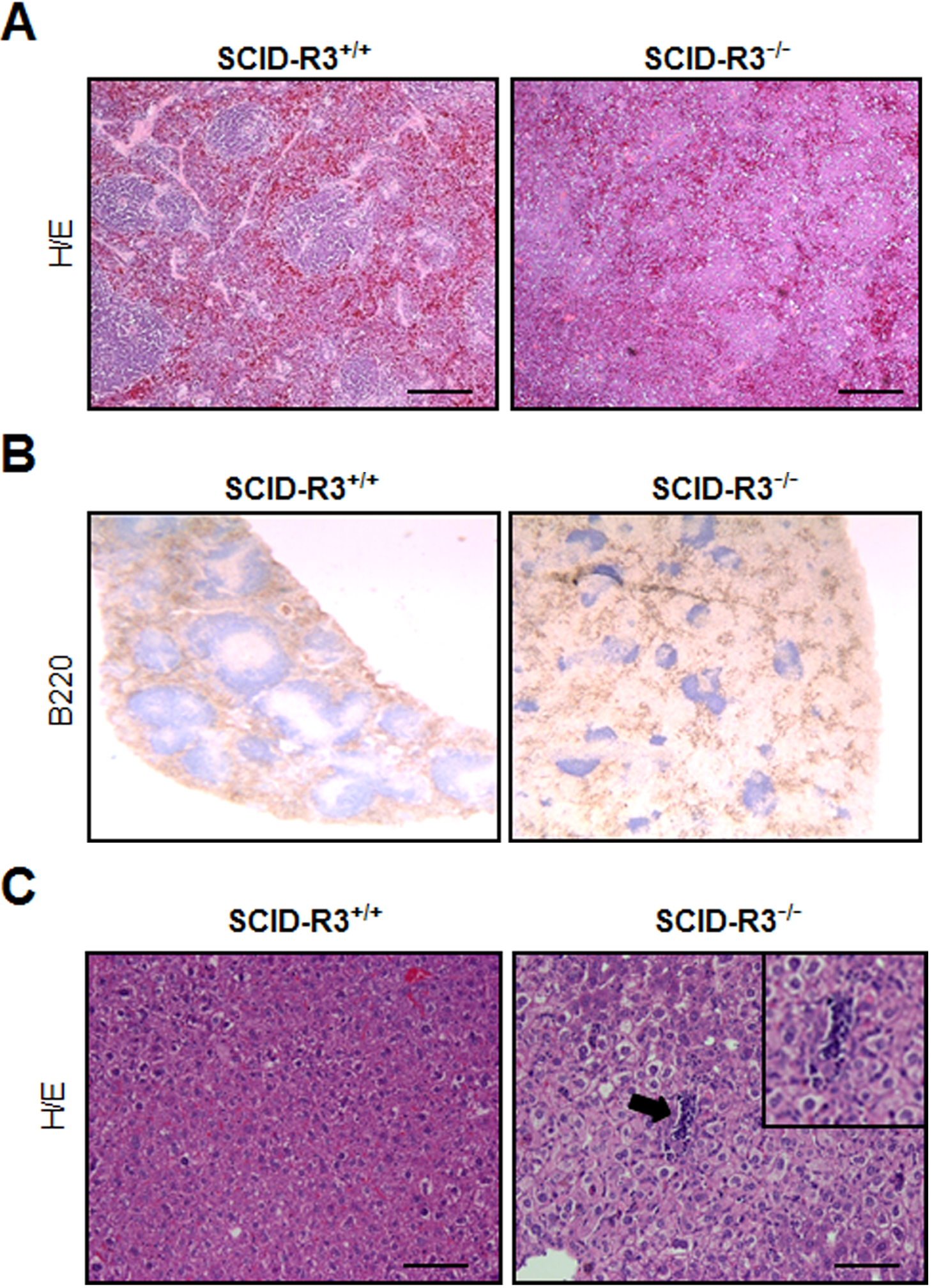

Supplement: Figure S2 — Abnormal splenic architecture and liver hematopoiesis in SCID-Rasa3−/− mice. Sections of age-matched SCID-Rasa3+/+ (left) and moribund SCID-Rasa3−/− (right) spleen were stained with (A) hematoxylin/eosin (H/E) or (B) a B220 antibody (B220). In SCID-Rasa3−/− spleen, the limits between red and white pulps are ill defined and the red pulp is infiltrated by cells of various sizes; the B cell compartment is also disorganized. C. Sections of age-matched SCID-Rasa3+/+ (left) and moribund SCID-Rasa3−/− (right) liver were stained with hematoxylin/eosin (H/E). Liver hematopoiesis (arrow) is observed in SCID-Rasa3−/− mice, but never in SCID-Rasa3+/+ mice. Inset: same image at higher magnification. (TIF) [file pgen.1004420.s002.tif]

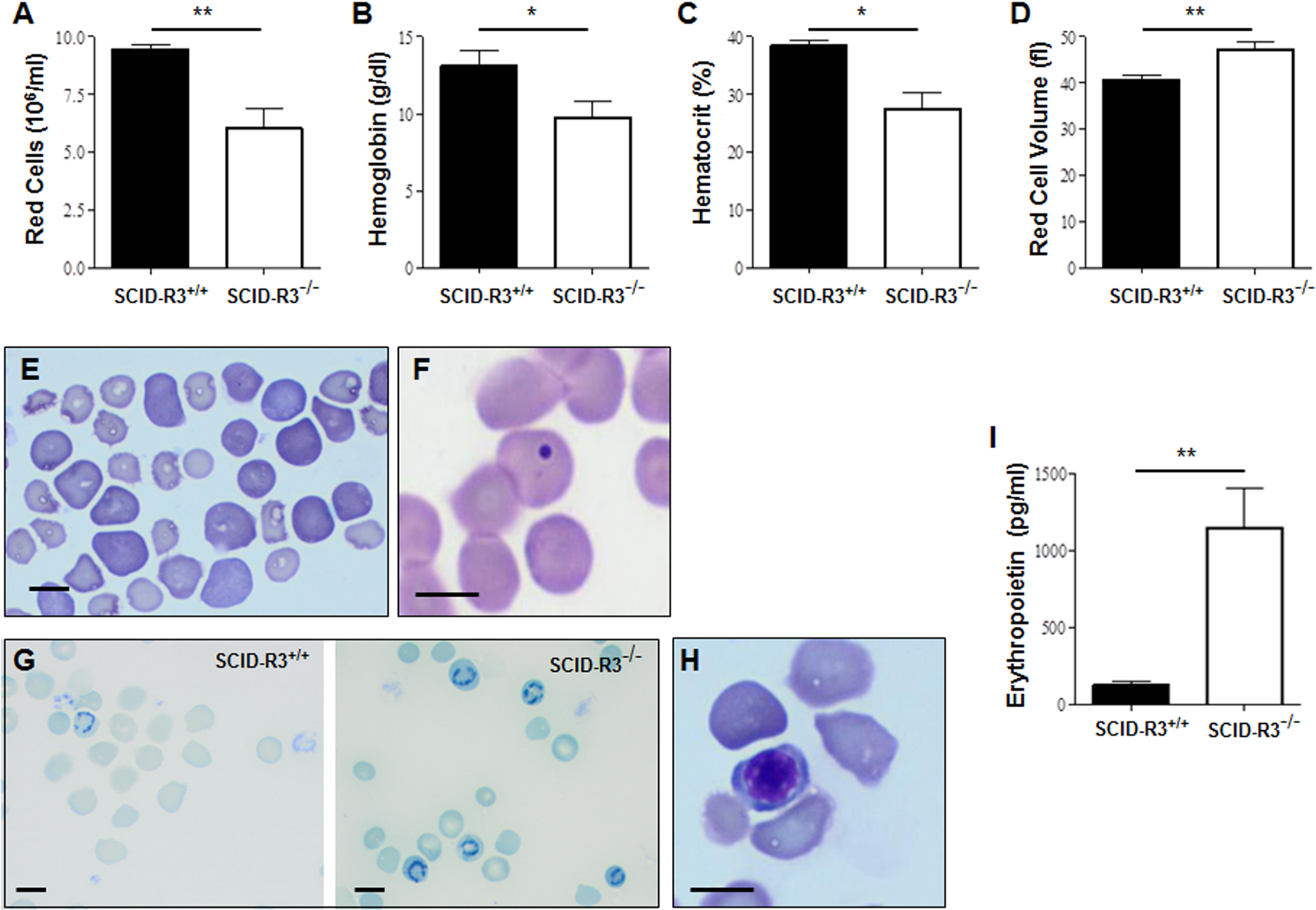

Supplement: Figure S3 — Regenerative anemia in SCID-Rasa3−/− mice. Blood analyses were performed on age-matched SCID-Rasa3+/+ and moribund SCID-Rasa3−/− mice. Mean ± SEM of red cell concentration (A), hemoglobin concentration (B), hematocrit (C) and red cell volume (D) in SCID-Rasa3+/+ (black columns, n = 7) and SCID-Rasa3−/− (white columns, n = 11) mice. Representative images of anisocytosis with polychromasia (E), of Howell-Jolly bodies (F), of increased reticulocytosis (G) and of metarubricytes (H) observed on blood smear from moribund SCID-Rasa3−/− mice. These alterations were not observed in age-matched SCID-Rasa3+/+ mice. I. Mean ± SEM of erythropoietin concentrations in age-matched SCID-Rasa3+/+ (black column, n = 8) and moribund SCID-Rasa3−/− (white column, n = 8) mice. Together, these alterations are classically associated with a regenerative anemia. Scale bars: 5 µm. Statistics (unpaired t test): *: P<0.05; **: P<0.01. (TIF) [file pgen.1004420.s003.tif]

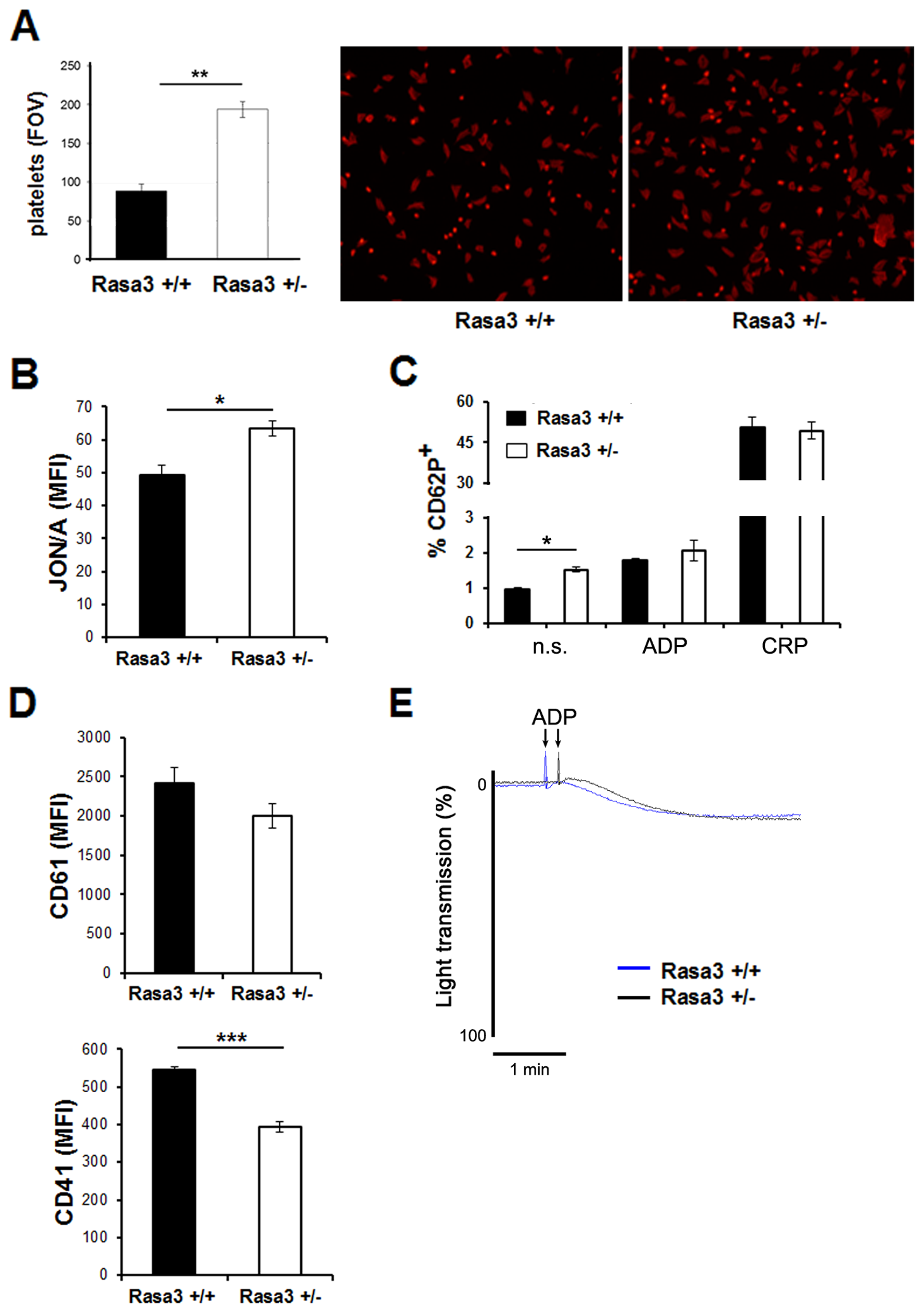

Supplement: Figure S4 — Altered platelet adherence and activation in adult Rasa3+/− mice. Unstimulated platelets were isolated from 8 week-old Rasa3+/+ and Rasa3+/− mice. A. After 45 min, an increased number of Rasa3+/− platelets adhered to BSA-coated plates, as compared with Rasa3+/+ platelets. Mean ± SEM of platelet counts per field of view (FOV) from two independent experiments performed in duplicate are represented. Statistics (unpaired t test): **: P<0.01. Representative images of Rasa3+/+ and Rasa3+/− adherent platelets after 45 min, stained with phalloidin-TRICT (actin, red). B. Mean ± SEM of the mean fluorescence intensity (MFI) of the JON/A antibody binding to the high affinity conformation of the integrin αIIbβ3 on Rasa3+/+ and Rasa3+/− platelets in resting condition. Results are representative of three separate experiments. Statistics (unpaired t test): *: P<0.05. C. Mean ± SEM of the percentage of CD62P+ platelets in non stimulated condition (n. s.) and after ADP (25 µM) or CRP (1 µg/ml) stimulation. Results are representative of three separate experiments. Statistics (unpaired t test): *: P<0.05. D. Mean ± SEM of the mean fluorescence intensity of CD61 and CD41 expression on Rasa3+/+ and Rasa3+/− platelets. Results are representative of three separate experiments. Statistics (unpaired t test): ***: P<0.001. E. Platelet aggregation assay revealed no aggregation defect in Rasa3+/− platelets in response to ADP (50 µM), as compared with Rasa3+/+ platelets. Results are representative of three separate experiments. (TIF) [file pgen.1004420.s004.tif]

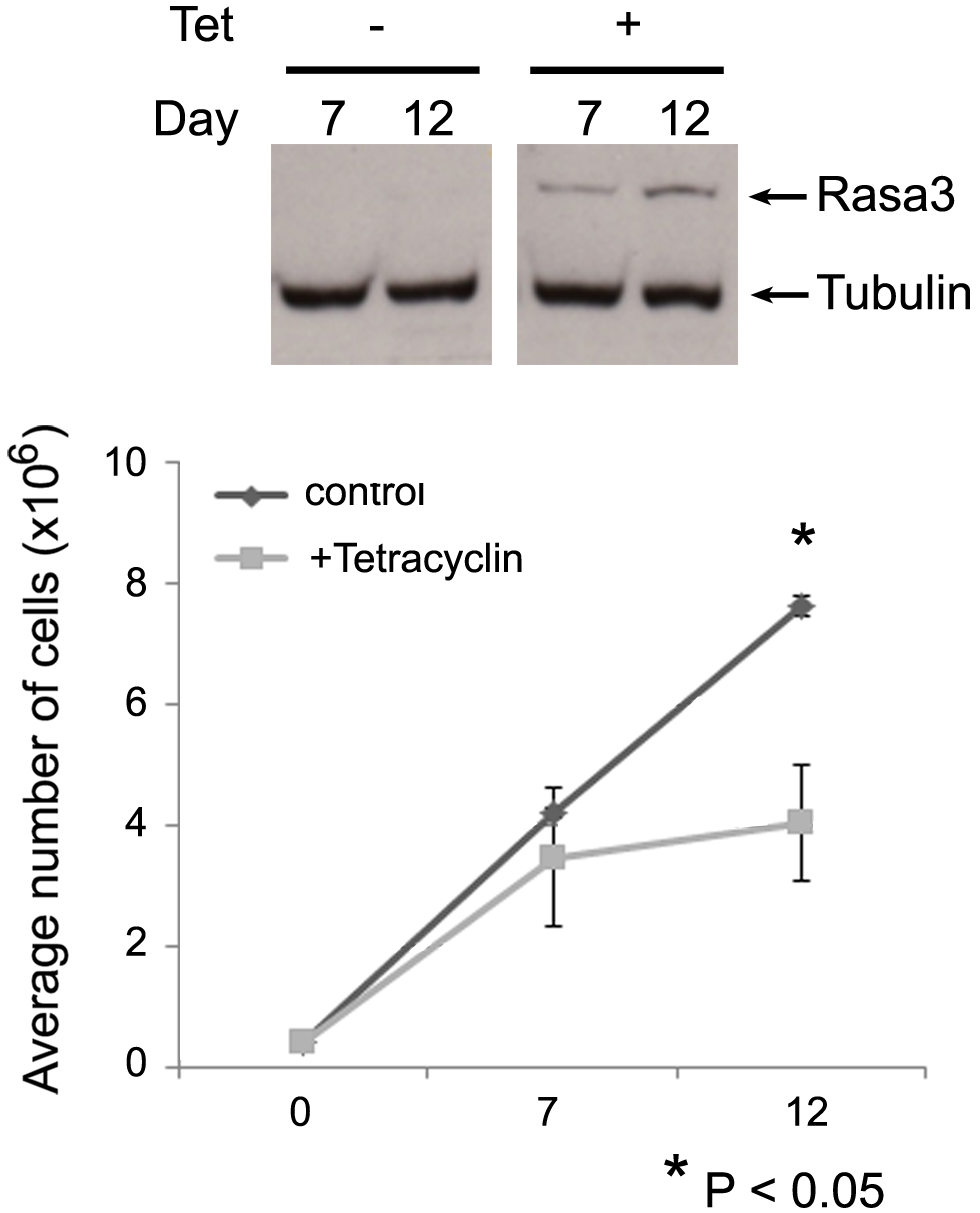

Supplement: Figure S5 — Effect of Rasa3 expression on K562 leukemic cell proliferation. Rasa3 expression in a mutant K562 leukemic cell line was induced by adding tetracycline in the culture medium for 12 days. At days 7 and 12, Rasa3 expression was analyzed by western blot and the number of living cells was measured with a hemocytometer. Graph represents the number of cells in the culture at days 7 and 12 (mean ± SEM, 3 independent experiments, each performed in duplicates/triplicates). Statistics (One-way anova): * P<0.05. (TIF) [file pgen.1004420.s005.tif]
