# Supplementary material for: Rasa3 Controls Megakaryocyte Rap1 Activation, Integrin Signaling and Differentiation into Proplatelet
Source: PLoS Genet. 2014 Jun 26;10(6):e1004420. doi: 10.1371/journal.pgen.1004420 (PMC4072513; doi:10.1371/journal.pgen.1004420)
Supplement: Table S1 — Total numbers of T and B cells were determined in the spleen of SCID-Rasa3+/+, SCID-Rasa3+/− and SCID-Rasa3−/− mice 6 weeks after irradiation/reconstitution by flow cytometry on the basis of 145-2C11 and B220 expression. A trend for higher B220+ B cell number was observed in SCID-Rasa3−/− mice as compared with SCID-Rasa3+/+ mice, but the difference did not reach statistical significance (P = 0.053, unpaired t test). Red blood cell, blood platelet and bone marrow megakaryocyte counts as well as spleen weight were also analyzed 6 weeks after irradiation/reconstitution. No significant difference was observed between SCID-Rasa3+/+ and SCID-Rasa3−/− mice. Megakaryocyte counts per field of view were obtained with a ×20 objective, 3 fields per mouse, 5 SCID-Rasa3+/+ and 4 SCID-Rasa3−/− mice. (DOC) [file pgen.1004420.s006.doc]

**Table S1: Splenic T and B cell, red blood cell, blood platelet and bone marrow megakaryocyte counts as well as spleen weight in SCID mice 6 weeks after irradiation/reconstitution:**

|  | **SCID-Rasa3^+/+^**  mean ± SEM  n = 3-5 | **SCID-Rasa3^+/-^**  mean ± SEM  n = 5 | **SCID-Rasa3^-/-^**  mean ± SEM  n = 3-5 |
| --- | --- | --- | --- |
| **CD3^+^ T cells** | 5.1 ± 0.5 x10^6^ | 6.4 ± 1.0 x10^6^ | 6.4 ± 1.5 x10^6^ |
| **B220^+^ B cells** | 6.4 ± 0.9 x10^6^ | 9.4 ± 2.2 x10^6^ | 15.3 ± 3.8 x10^6^ |
| **Red blood cells** | 9.4 ± 0.7 x 10^6^/ml |  | 9.0 ± 1.3 x 10^6^/ml |
| **Blood platelets** | 612 ± 133 x 10^3^/µl |  | 678 ± 159 x 10^3^/µl |
| **Bone marrow**  **Megakaryocytes (n per field of view)** | 5.6 ± 0.6 |  | 5.7 ± 0.3 |
| **Spleen weight** | 75 ± 8 mg |  | 79 ±10 mg |
|  |  |  |  |
