# Supplementary material for: Rasa3 Controls Megakaryocyte Rap1 Activation, Integrin Signaling and Differentiation into Proplatelet
Source: PLoS Genet. 2014 Jun 26;10(6):e1004420. doi: 10.1371/journal.pgen.1004420 (PMC4072513; doi:10.1371/journal.pgen.1004420)
Supplement: Table S2 — Bone marrow cells were isolated from SCID-Rasa3+/+ and SCID-Rasa3−/− mice 2 months after irradiation/reconstitution, incubated with antibodies directed against cell surface markers and analyzed by flow cytometry for the percentage of cells within the bone marrow cells or within a subpopulation of bone marrow cells defined by specific markers. (DOC) [file pgen.1004420.s007.doc]

**Table S2: Flow cytometry analysis of bone marrow hematopoietic stem and megakaryocyte progenitor cells in SCID-Rasa3 mice:**

| **Bone marrow cells** | **SCID-Rasa3^+/+^**  mean ± SEM (%)  (n = 4) | **SCID-Rasa3^-/-^**  mean ± SEM (%)  (n = 4) | **Statistics**  (unpaired *t* test) |
| --- | --- | --- | --- |
| c-Kit^+^ | 6.9 ± 3.5 | 8.8 ± 3.9 | P>0.05 |
| c-Kit^+^/Lin^-^ | 1.95 ± 0.05 | 2.03 ± 0.07 | P>0.05 |
| Sca-1^+^ within the c-Kit^+^ Lin^-^ population | 21.0 ± 2.1 | 22.8 ± 3.5 | P>0.05 |
| **Hematopoietic Stem Cells:**  CD34^-^ Flk-2^-^ within the c-Kit^+^ Lin^-^ Sca-1^+^ population | 7.9 ± 1.6 | 8.9 ± 2.1 | P>0.05 |
| FcγRII/III^lo^  Sca-1^-^ within the c-Kit^+^ Lin^-^ population | 2.9 ± 0.7 | 2.6 ± 0.7 | P>0.05 |
| **Megakaryocyte progenitor cells:**  CD150^+^ within the FcγRII/III^lo^  Sca-1^-^ c-Kit^+^ Lin^-^ population | 35.5 ± 5.0 | 30.1 ± 3.4 | P>0.05 |
