# Supplementary material for: Rasa3 Controls Megakaryocyte Rap1 Activation, Integrin Signaling and Differentiation into Proplatelet
Source: PLoS Genet. 2014 Jun 26;10(6):e1004420. doi: 10.1371/journal.pgen.1004420 (PMC4072513; doi:10.1371/journal.pgen.1004420)
Supplement: Table S3 — Age-matched SCID-Rasa3+/+ and moribund SCID-Rasa3−/− mice were analyzed for their total number of nucleated splenocytes and, after flow cytometry with relevant antibodies, for their percentages (%) and cell numbers (n) of splenic mature (macrophages, T and B cells) and immature (megakaryocytes, myeloid cells, hematopoietic progenitors and erythroblasts) cells. Results indicate that in SCID-Rasa3−/− mice, total number of nucleated splenocytes as well as percentage and number of immature splenic cells are significantly increased, consistent with a markedly increased hematopoiesis in the spleen of these mice, as compared with SCID-Rasa3+/+ mice. By contrast, the percentage of mature cells is decreased in the spleen of SCID-Rasa3−/− mice, as compared with SCID-Rasa3+/+ mice, although their number is increased, a probable consequence of the increased hematopoiesis in this organ. (DOC) [file pgen.1004420.s008.doc]

**Table S3: Increased splenic hematopoiesis in SCID-Rasa3^-/-^** mice:

|  |  | **SCID-Rasa3^+/+^**  mean ± SEM  (12<n<17) | **SCID-Rasa3^-/-^**  mean ± SEM  (19<n<24) | **Statistics**  (unpaired *t* test) |
| --- | --- | --- | --- | --- |
| **Nucleated splenocytes** | n | 78.6 ± 11.8 x 10^6^ | 367.8 ± 83.4 x 10^6^ | P<0.001 |
| **F4.80^+^ macrophages** | %  n | 7.6 ± 0.9  6.0 ± 0.7 x 10^6^ | 4.1 ± 0.9  15.1 ± 3.3 x 10^6^ | P<0.001  P<0.001 |
| **CD3^+^ T cells** | %  n | 24.7 ± 1.5  19.4 ± 1.2 x 10^6^ | 13.3 ± 1.2  48.9 ± 4.4 x 10^6^ | P<0.001  P<0.001 |
| **B220^+^ B cells** | %  n | 49.4 ± 1.2  38.8 ± 0.9 x 10^6^ | 28.7 ± 3.9  105.6 ± 14.3 x 10^6^ | P<0.001  P<0.001 |
| **CD41^+^ megakaryocytes** | %  n | 1.5 ± 0.4  1.2 ± 0.3 x 10^6^ | 4.0 ± 0.5  14.7 ± 1.8 x 10^6^ | P<0.05  P<0.001 |
| **Gr1^int^ Mac1^+^ myeloid cells** | %  n | 3.4 ± 0.2  2.7 ± 0.2 x 10^6^ | 5.7 ± 0.7  21.0 ± 2.6 x 10^6^ | P<0.05  P<0.001 |
| **CD117^+^ hematopoietic progenitors** | %  n | 9.0 ± 3.9  7.0 ± 3.1 x 10^6^ | 33.9 ± 3.2  124.7 ± 11.8 x 10^6^ | P<0.01  P<0.001 |
| **Ter119^+^ CD71^+^ erythroblasts** | %  n | 2.1 ± 0.5  1.7 ± 0.4 x 10^6^ | 9.2 ± 1.8  33.8 ± 6.6 x 10^6^ | P<0.001  P<0.001 |
