# Supplementary material for: Rasa3 Controls Megakaryocyte Rap1 Activation, Integrin Signaling and Differentiation into Proplatelet
Source: PLoS Genet. 2014 Jun 26;10(6):e1004420. doi: 10.1371/journal.pgen.1004420 (PMC4072513; doi:10.1371/journal.pgen.1004420)
Supplement: Table S4 — Age-matched SCID-Rasa3+/+ and moribund SCID-Rasa3−/− mice were analyzed for their total number of white cells and circulating neutrophils, lymphocytes, monocytes and eosinophils on Giemsa-stained blood smears. (DOC) [file pgen.1004420.s009.doc]

**Table S4: White blood cell and circulating neutrophil, lymphocyte, monocyte and eosinophil counts in SCID-Rasa3^+/+^ and SCID-Rasa3^-/-^ mice:**

|  | **SCID-Rasa3^+/+^**  mean ± SEM  (n = 6) | **SCID-Rasa3^-/-^**  mean ± SEM  (n = 5) | **Statistics**  (unpaired *t* test) |
| --- | --- | --- | --- |
| **White blood cells** | 6.2 ± 1.0 x 10^9^/l | 4.7 ± 1.4 x 10^9^/l | P>0.05 |
| **Neutrophils** | 1.1 ± 0.3 x 10^9^/l | 1.0 ± 0.3 x 10^9^/l | P>0.05 |
| **Lymphocytes** | 4.5 ± 0.9 x 10^9^/l | 3.5 ± 1.1 x 10^9^/l | P>0.05 |
| **Monocytes** | 0.6 ± 0.1 x 10^9^/l | 0.5 ± 0.1 x 10^9^/l | P>0.05 |
| **Eosinophils** | 0.03 ± 0.001 x 10^9^/l | 0.02 ± 0.001 x 10^9^/l | P>0.05 |
