# Supplementary material for: Rasa3 Controls Megakaryocyte Rap1 Activation, Integrin Signaling and Differentiation into Proplatelet
Source: PLoS Genet. 2014 Jun 26;10(6):e1004420. doi: 10.1371/journal.pgen.1004420 (PMC4072513; doi:10.1371/journal.pgen.1004420)
Supplement: Table S5 — Genetic, biological and phenotypical differences between Rasa3Scat/Scat, Rasa3−/− and SCID-Rasa3−/− mice. (DOC) [file pgen.1004420.s010.doc]

**Table S5: Genetic, biological and phenotypical differences between Rasa3^Scat/Scat^, Rasa3^-/-^ and SCID-Rasa3^-/-^ mice:**

|  | **Rasa3^Scat/Scat^**  [18] | **Rasa3^-/-^**  [16] | **SCID-Rasa3^-/-^** |
| --- | --- | --- | --- |
| **Genetic alteration and its consequence** | - Spontaneous  - Missense mutation (G125V) in the exon 5 coding region, between the two C2 domains of Rasa3.  - Relocalization of Rasa3 protein from the membrane to the cytosol. | - Induced  - Replacement of exons 11 and 12, coding for an essential region of the catalytic activity, by a neomycine resistance cassette.  - Production of a catalytically-inactive truncated Rasa3 protein. | - Idem Rasa3^-/-^  - Idem Rasa3^-/-^  - Idem Rasa3^-/-^ |
| **Genetic background** | BalB/cBy | Hybrid C57BL/6J x 129/SvJ | Hybrid C57BL/6J x 129/SvJ |
| **Localization of the genetic alteration** | Ubiquitous, but the full phenotype is transferable via hematopoietic stem cells | ubiquitous | Hematopoietic stem cells only |
| **Survival** | Cyclic phenotype of crisis-remission: first embryonic-P9 wave of lethality (60% of mutant mice) and second P30 wave of lethality (94% of the survivors) | Embryonic lethal (E12.5-13.5): 100% | Progressive lethality over 14 months after SCID mice reconstitution |
| **Mechanism of anemia** | Delayed erythropoiesis with accumulation of polychromatic and orthochromatic erythroblasts in the spleen, but not in the bone marrow, associated with increased GTP-bound Ras |  | Hallmarks of regenerative anemia ( i. e. presence of a red cell anisocytosis with polychromasia, of Howell-Jolly bodies, of an increased reticulocytosis and, sometimes, of metarubricytes), most probably due to hemorrhages |
| **Bone marrow fibrosis** | Not reported |  | + |
| **Extra-medullar hematopoiesis** | Not reported |  | + (spleen and liver) |
| **Mechanism of thrombocytopenia** | Unknown |  | constitutive activation of inside-out and outside-in integrin signaling in megakaryocytes caused by increased active GTP-bound Rap1 level, leading to cell adherence and spreading independently of integrin ligands, altered actin cytoskeletal organization, reduced motility and altered repartition of megakaryocytes in osteoblastic and vascular niches. |
| **Oncogenesis** | Negative |  | preleukemia (~20% of mice) |
